# An O_2_-sensing diguanylate cyclase broadly affects the aerobic transcriptome in the phytopathogen *Pectobacterium carotovorum*

**DOI:** 10.3389/fmicb.2023.1134742

**Published:** 2023-07-07

**Authors:** Florian J. Fekete, Nick J. Marotta, Xuanyu Liu, Emily E. Weinert

**Affiliations:** ^1^Department of Biochemistry and Molecular Biology, Penn State University, University Park, PA, United States; ^2^Graduate Program in Molecular, Cellular, and Integrative Biosciences, Penn State University, University Park, PA, United States; ^3^Department of Chemistry, Penn State University, University Park, PA, United States

**Keywords:** cyclic-di-GMP, oxygen, *Pectobacterium*, globin, diguanylate cyclase

## Abstract

*Pectobacterium carotovorum* is an important plant pathogen responsible for the destruction of crops through bacterial soft rot, which is modulated by oxygen (O_2_) concentration. A soluble globin coupled sensor protein, *Pcc* DgcO (also referred to as *Pcc*GCS) is one way through which *P. carotovorum* senses oxygen. DgcO contains a diguanylate cyclase output domain producing c-di-GMP. Synthesis of the bacterial second messenger c-di-GMP is increased upon oxygen binding to the sensory globin domain. This work seeks to understand regulation of function by DgcO at the transcript level. RNA sequencing and differential expression analysis revealed that the deletion of DgcO only affects transcript levels in cells grown under aerobic conditions. Differential expression analysis showed that DgcO deletion alters transcript levels for metal transporters. These results, followed by inductively coupled plasma—mass spectrometry showing decreased concentrations of six biologically relevant metals upon DgcO deletion, provide evidence that a globin coupled sensor can affect cellular metal content. These findings improve the understanding of the transcript level control of O_2_-dependent phenotypes in an important phytopathogen and establish a basis for further studies on c-di-GMP-dependent functions in *P. carotovorum*.

## 1. Introduction

Bacteria sense and respond to environmental signals such as nutrient availability, temperature, signals from other organisms, and oxygen (O_2_) (Miller and Bassler, 2001; Toth and Birch, 2005; Charkowski, 2006). Obligate and facultatively aerobic organisms can use molecular O_2_ as a terminal electron acceptor, while many obligate anaerobe species are inhibited by its presence (Taylor, 1983). O_2_ availability can influence numerous bacterial functions, such as motility, virulence factor expression, and host colonization (Miller and Bassler, 2001; Edwards et al., 2006; Walker et al., 2017). O_2_ can be sensed directly by ligand binding or indirectly through redox sensors (Edwards et al., 2006; Vinogradov et al., 2013). Globin coupled sensor (GCS) proteins are a wide-spread family of direct gas sensing proteins that have been identified in hundreds of bacterial genomes and are also predicted in some archaea and lower eukaryotes (Vinogradov et al., 2013; Walker et al., 2017). GCS proteins consist of a heme-containing globin domain, which binds diatomic ligands (O_2_, NO, CO) at the iron center, linked by a variable middle domain to a variety of output domains, such as phosphodiesterases, methyl-accepting chemotaxis proteins, adenylate cyclases, and diguanylate cyclases (Vinogradov et al., 2013; Walker et al., 2017).

Commonly found in prokaryotes, GGDEF domains are responsible for synthesizing the second messenger bis-(3′-5′)-cyclic diguanylate (c-di-GMP) (Römling et al., 2013; Jenal et al., 2017; Hengge, 2021), which controls functions such as motility, virulence, and biofilm formation (Boyd and O'Toole, 2012; Jenal et al., 2017; Hengge, 2021). C-di-GMP levels can regulate cellular functions by directly interacting with target proteins or through regulating the activity of riboswitches (Sudarsan et al., 2008; Boyd and O'Toole, 2012; Jenal et al., 2017; Hengge, 2021). Evidence suggests that, besides the overall cellular c-di-GMP pool, local concentrations of c-di-GMP also affect protein activity, and thus cellular function, in a specific, localized manner (Hengge, 2021). These interactions can involve GGDEF proteins closely interacting with their targets, tightly regulating local c-di-GMP concentrations and therefore protein activity (Hengge, 2021). While numerous targets and ways of regulation have been identified, the full extent of bacterial c-di-GMP signaling remains uncharacterized (Hengge, 2010; Boyd and O'Toole, 2012; Römling et al., 2013; Jenal et al., 2017; Hengge, 2021).

The phytopathogen *Pectobacterium carotovorum* subsp. *carotovorum* (*Pcc*) is a leading cause of bacterial soft rot and is responsible for crop losses worth millions of US dollars each year (Toth and Birch, 2005; Charkowski, 2006; Kim et al., 2009). Upon infecting the plant host, *Pcc* secretes plant cell wall degrading exoenzymes (PCWDEs); this varied group of pectate lyases, cellulases, and polysaccharide lyases is responsible for the rotting of plant tissue (Toth and Birch, 2005; Charkowski, 2006; Babujee et al., 2012; Põllumaa et al., 2012). *Pcc* is known to exhibit increased virulence under low oxygen concentrations, which is often caused by flooding of fields (Babujee et al., 2012). *Pcc* encodes for one GCS protein, previously known as *Pcc*GCS, which we hereby propose to rename *Pcc*DgcO based on the conserved GGDEF motif and the previously established systematic nomenclature of *E. coli* GGDEF domain-containing proteins and based on the similarity of *Pcc*GCS to the *E. coli* GCS protein *Ec*DgcO (also known as *Ec*DosC) (Tuckerman et al., 2009; Burns et al., 2014; Tarnawski et al., 2015; Hengge et al., 2016; Burns et al., 2017; Walker et al., 2017). Biochemical characterization of *Pcc*DgcO was demonstrated diguanylate cyclase activity in response to O_2_ binding to the sensor globin and a number of phenotypes associated with *Pcc*DgcO have been studied in the *Pcc* strain WPP14 (Kim et al., 2009; Burns et al., 2014, 2017; Walker et al., 2017; Rivera et al., 2018). *Pcc*DgcO is known to regulate virulence, motility and extracellular levels of the quorum sensing molecule N-acyl homoserine lactone (AHL) under aerobic conditions, but has not been associated with phenotypes anaerobically (Miller and Bassler, 2001; Burns et al., 2017).

Transcriptomics, proteomics, phenotypic assays, and inductively coupled plasma-mass spectrometry (ICP-MS) were used to investigate the full role of *Pcc*DgcO in regulating cellular function. Results of these studies provide new insights into how *Pcc*DgcO regulates previously described phenotypes, identifies additional protein interactors of *Pcc*DgcO, and provides evidence of *Pcc*DgcO-dependent control of cellular metal homeostasis. Overall, this work provides insight into the *Pcc*DgcO-dependent regulation of cellular oxygen response in *P. carotovorum.*

## 2. Materials and methods

### 2.1. Bacterial strains and general information

Wild-type (WT) *Pcc* WPP14 and the *Pcc*DgcO deletion strain (Δ*dgcO*) strains were used in all assays, unless otherwise noted. Construction of the Δ*dgcO* strain was performed by Creative Biogene, Inc. (Shirley, NY, United States). Briefly, the *dgcO* gene was PCR amplified from WT *Pcc* and cloned into a pLP12 suicide vector conferring resistance to chloramphenicol (Luo et al., 2015), and transformed into *E. coli* DH5α cells. Plasmids extracted from positive clones were then electroporated into *E. coli* strain β2163 cells, which was then used to conjugate the deletion sequence into WT *Pcc* cells to delete the *dgcO* gene through homologous recombination. The deletion region was then verified by PCR amplification of the target sequence, followed by sequencing to verify the presence of a scar sequence (Supplementary file S1), as commonly used in the literature (Mole et al., 2010; Marquez-Villavicencio et al., 2011; Bowden et al., 2013; Koiv et al., 2013; Ernst et al., 2020). Primer sequences for amplification and sequencing of deletion region (5′ → 3′): GATCGCCATCAGTGCCAAGG (anneals bp. 1781101–1781120), CATCGTTGGAAGAGCAAGCAG (anneals bp. 1783954–1783974). Primers annealing within *dgcO* were designed and used to verify the knockout (5′ → 3′): GAACGATCATGAATTC (anneals bp. 1782149–1782164), GCTGACGCGTTTTAC (anneals bp. 1783158–1783172). All coordinate references from the published *Pcc* WPP14 genome under NCBI GenBank accession number CP051652.1 with the primary assembly GCF_013488025 (Liu et al., 2020). Additionally, the deletion and the lack of further insertions was verified using 200 Mbp Illumina whole genome sequencing by SeqCenter, LLC (Pittsburgh, PA, United States).

All chemicals and reagents were purchased and used without further purification.

For all assays, bacteria were grown on Luria-Bertani (LB) (Research Products International) agar (Sigma-Aldrich) plates at 30°C, isolated colonies from these plates were used to inoculate liquid cultures specified in each experiment. Data represents *n* = 3 biological replicates, unless stated otherwise. Statistical analyses were performed as described at each experiment, with a *p-*value < 0.05 being considered significant for all assays.

### 2.2. RNA sequencing and differential expression analysis

To quantify mRNA levels, WT and Δ*dgcO Pcc* were grown overnight on Luria-Bertani (LB) (RPI) agar plates. Three isolated colonies of each strain were inoculated into 3 mL LB broth and grown overnight at 30°C with 225 rpm shaking in 15 mL plastic culture tubes (VWR). LB was chosen as *Pcc* grows well in LB at 30°C, and as it has been used for transcriptomic analyses for *Pectobacterium* species in the past (Gorshkov et al., 2017; Wang et al., 2018; Fan et al., 2020). From these cultures, 50 μL was inoculated into 5 mL fresh LB and grown at 30°C with 225 rpm orbital shaking in 15 mL culture tubes either aerobically (grown to OD_600_ ~ 0.68) or anaerobically in an anaerobic glovebag (Coy; grown to OD_600_ ~ 0.2). RNA extraction for all cultures was performed identically, under aerobic conditions. 1 mL of each culture was pelleted and then RNA extraction was performed using a New England Biolabs Monarch RNA Miniprep Kit (T2010S), according to the procedures outlined by the manufacturer. RNA samples were flash-frozen in liquid N_2_ and stored at −80°C until submission for analysis by SeqCenter, LLC (Pittsburgh, PA) on dry ice, where RNA sequencing and differential expression analysis was performed. Briefly, Illumina Stranded RNA library preparation with RiboZero Plus rRNA depletion was performed, with 25 M reads. Quality control and adapter trimming was performed with bcl2fastq (version: 2.20.0.445 with default parameters) (Illumina, 2019). Read mapping was performed with HISAT2 (version: 2.2.0 with default parameters + “--very-sensitive”) (Kim et al., 2019). Read quantification was performed using Subread’s featureCounts (version: 2.0.1 with default parameters + “-Q 20”) functionality (Liao et al., 2014). Read counts were loaded into R (version: 4.0.2 with default parameters) and were normalized using edgeR’s (version: 1.14.5 with default parameters) Trimmed Mean of M values (TMM) algorithm (Robinson et al., 2010). Subsequent values were then converted to counts per million (cpm). Differential expression analysis was performed using edgeR’s Quasi Linear *F*-test. Significance of differential expression was established by using |log_2_FC| > 1 and adjusted *p*-values of < 0.05, calculated by the Benjamini-Hochberg method, with the false detection rate being <0.05. The NCBI GenBank accession number CP051652.1 with the primary assembly GCF_013488025.1 was used to annotate the genomes and perform the analyses (Liu et al., 2020). The *dgcO* gene is annotated by the LocusTag HER17_08095. Annotated transcripts were manually classified in Microsoft Excel using online tools, such as the NCBI Gene database, NCBI BLAST, and Uniprot (Altschul et al., 1990; Doǧan et al., 2016). Sequencing data was submitted to the NCBI Gene Expression Omnibus (GEO) under the accession: GSE214075.

### 2.3. Quantification of c-di-GMP

WT and Δ*dgcO Pcc* were grown overnight at 30°C on LB agar plates. Three isolated colonies of each strain were inoculated into 3 mL LB broth overnight at 30°C with 225 rpm shaking in 15 mL plastic culture tubes (VWR). For quantification of c-di-GMP from aerobically growing *Pcc*, overnight cultures were diluted 1:100 into 15 mL LB in 50 mL plastic culture tubes (Falcon) and grown at 30°C with 225 rpm shaking to an OD_600_ of ~0.68. For quantification of c-di-GMP produced anaerobically, the overnight cultures were diluted 1:100 into 15 mL LB in 50 mL conicals (Falcon) in an anaerobic glovebag (Coy). Cultures were grown at 30°C with 230 rpm shaking to OD_600_ ~ 0.18. All further steps were performed aerobically for all cultures. 10 mL of each culture were pelleted at 3,000°×°g for 15 min at 4°C, the supernatant decanted, the pellets flash frozen in liquid N_2_ and stored at −80°C until use. Extraction of c-di-GMP was done as described previously, with minor modifications (Fontaine et al., 2018). Pellets were resuspended in 1 mL cold extraction solvent/5 mL of original culture, consisting of 2/2/1 v/v/v MeCN/MeOH/H_2_O. Resuspended cells were then lysed by sonication in a cup horn (Qsonica Q500, part #431C2) at 85% amplitude, 15 s on/15 s off, for a total sonication time of 6 min. Sonicated samples were then centrifuged at 3,500 × g for 10 min at 4°C to pellet debris. After centrifugation, supernatants were dried by lyophilization and stored at −80°C until analysis by mass spectrometry (MS). Prior to analysis, samples were resuspended in 250 μL HPLC grade water containing 0.5 μM 8-bromoadenosine 3′,5′-cyclic monophosphate (8-Br 3′,5′-cAMP) (Millipore Sigma) as an internal standard and centrifuged at 12,000 × g at 4°C for 15 min to further remove debris. 50 μL of supernatant was then submitted for analysis to the Penn State Metabolomics Core Facility (University Park, PA, United States) in LC–MS autosampler vials.

Samples were analyzed by Vanquish UHPLC system coupled with a TSQ Quantis Plus mass spectrometer in positive ion mode using a H-ESI^™^ ion source (all Thermo Fisher Scientific) with a Waters (Milford, MA) CORTECS C18 + column (2.1 × 100 mm, 1.6 um particle size). The injection volume was 2 μL. The mobile phase was composed of HPLC grade water with 0.1% formic acid (solvent A) and/or acetonitrile with 0.1% formic acid (solvent B). The flow rate of the mobile phase was 0.3 mL/min with column temperature of 40°C. The solvent gradient was as follows: 0% B from 0 to 4 min, 1.5% B from 4 to 15 min, 15% B from 20 to 25 min, increasing to 85% B over 25 to 28 min, then 100% B from 28 to 35 min. Mobile phase was held at 100% B until 39 min, then switched to 0% B (100% A) until 50 min. During the analysis, an ion spray voltage of 4,000 V, sheath gas of 40 psi, aux. Gas of 12 psi, sweep gas of 1, ion transfer tube temperature of 325°C and vaporizer temperature of 300°C were applied. C-di-GMP was detected based on a precursor m/z of 691.135 with fragments at 135.21 (collision energy [CE] 90 V), 152.118 (CE 60 V), and 248.168 (CE 40 V). 8-Br 3′,5′-cAMP was detected based on a precursor of 408.03 m/z and fragments of 134.92 (CE 53 V), and 213.83 (CE 25 V).

Peak integration was performed using Freestyle version 1.8.63.0. For c-di-GMP, retention time and fragmentation were compared to an authentic standard and the fragment at 152.118 m/z was used for quantification. For 8-Br 3′,5′-cAMP, 213.83 was used for quantification. C-di-GMP peak area was divided by 8-Br 3′,5′-cAMP peak area to normalize for ionization efficiency between samples. This peak area ratio was then normalized to cell pellet mass.

### 2.4. Aerobic to anaerobic growth transition

To assess transition from aerobic to anaerobic growth, *Pcc* WT and Δ*dgcO* were grown overnight on LB agar plates at 30°C. Four isolated colonies of each strain were inoculated into 3 mL LB broth in 15 mL plastic culture tubes (VWR), and grown overnight at 30°C with 225 rpm shaking. 980 μL LB or M9 + LLL media were added to each well of a 24 well plate, and the overnight cultures were diluted in fresh LB and were added to the wells to a normalized 0.005 OD_600_ starting concentration (~20 μL for each well). Plates were then covered by BreathEasy^™^ (Diversified Bioscience) film and placed into a Biotek Epoch 2 plate reader for 315 min at 30°C with 228 cpm continuous orbital shaking. OD_600_ was measured every 15 min. After 315 min, the plate was removed from the plate reader, and holes were poked above each well on the film using a sterile needle. The lid was placed back onto the plate, and the plate was then cycled into an anaerobic chamber (Coy). After cycling into the chamber, the lid was removed and the plate was covered with a layer of film to prevent contamination and evaporation. The plate was then placed into a Biotek Epoch 2 plate reader and grown anaerobically for 24 h under identical parameters to the aerobic portion of this assay.

To assess transition from anaerobic to aerobic growth, overnight cultures of WT and Δ*dgcO Pcc* were inoculated in an identical way as for measuring aerobic to anaerobic transition, except that the overnight cultures were set up in an anaerobic chamber (Coy), in de-gassed LB media. Layout and preparation of 24 well plate was all performed in the glovebag, with steps identical to aerobic to anaerobic transition. The plate was placed into a Biotek Epoch 2 plate reader for 360 min at 30°C with 228 cpm continuous orbital shaking. OD_600_ was measured every 15 min. The plate was then cycled out of the chamber and placed into a Biotek Epoch 2 plate reader and grown aerobically for 24 h under identical parameters to the anerobic portion of the assay.

After the growths were completed, means and standard deviations for each strain were calculated, and the OD_600_ values were plotted as a function of time in Igor Pro 6.10.

### 2.5. Identification of proteins interacting with *Pcc*DgcO

Pull-down and identification of proteins interacting with *Pcc*DgcO was performed as previously described (Burns et al., 2017). Briefly, *Pcc*DgcO was expressed and purified as previously described (Burns et al., 2014). WT *Pcc* WPP14 5 mL overnight LB cultures were grown at 30°C in a shaking incubator and then spun down using an Allegra X-30R centrifuge. Novagen BugBuster^®^ Protein Extraction Reagent protocol was then followed to lyse the cell pellets. Total cell lysate was then spun down at 12,000 × g and supernatant (~200–500 μL) mixed with an equal amount of purified Fe^II^-O_2_ *Pcc* DgcO, and mixed using an orbital mixer for 2 h at 4°C. Nickel-NTA resin was prepared using a 20 mM imidazole buffer (50 mM Tris, 20 mM Imidazole, 300 mM NaCl, 5% glycerol, pH = 7.0). After mixing for 2 h, the mixture was loaded onto 1 mL of prepared resin and flow through was collected. Then 1 mL of 20 mM imidazole buffer was added, allowed to drip through the column, and collected as the wash. In 1 mL increments, the buffers containing increasing amounts of imidazole (50, 100, and 200 mM imidazole buffers) were loaded onto the resin and flow-through collected. All fractions were then flash frozen in liquid N_2_ and stored at −80°C until used for protein digestion. The pull-downs were repeated twice, with one biological replicate in the first run and three biological replicates in the second run.

Tryptic and chymotryptic digestions were performed on each fraction using the In-solution Tryptic Digestion and Guanidination Kit (Thermo Scientific) and Mass Spectrometry-Grade Chymotrypsin Endoproteinase (Thermo Scientific) according to their respective kit’s instructions. In brief, 10.5 μL of each fraction was mixed with the respective digestion buffer. Samples were heated at 95°C, cooled, alkylated using iodoacetamide (IAA), and then 1 μL of the respective protease was added to the mixture and allowed to react overnight at room temperature before 3 μL of trifluoroacetic acid (TFA) was added to stop the reaction. Digestion samples were loaded into mass spectrometer vials.

The mass spectrometry experiments were performed on an LTQ-FTMS Ultra from Thermo Fisher equipped with a Thermo Ultimate 3,000 HPLC dual pump controlled through Chromeleon software and a Shimadzu SIL-ACHT autosampler controlled by a Shimadzu CBM-20A controller. The control of the mass spectrometer and the synchronization of the system was accomplished in the Xcalibur software supplied by Thermo Fisher.

One of the dual pumps was used to load the sample injected from the autosampler onto a Micro Bioresource Captrap and washed using 2% formic acid in LC/MS water at a flow rate of 50 μL/min for 20 min. The Captrap was switched in the flow from the second pump into the picochip [New Objective (1PCH7515-105H354-NV)] using the divert valve loaded on to the capillary. The flow from the pump to the chip was set at 0.150 mL/min but was split using a homemade splitter just before the diverter valve; based on pressure obtained and specifications of the microchip, it is estimated that the flow rate to the chip was approximately 1 μL/min. The chromatography profile was as follows: 98% A (0.1% formic acid in H_2_O) and 2% B (0.01% formic acid in acetonitrile) for the 20 min; followed by a gradient to 60% A/40% B over 90 min; followed by a gradient to 2% A/98% B over 40 min; followed by a gradient to 98% A/2% B over 40 min.

The LTQ FTMS Ultra acquired a full scan FTMS from 200 to 2,000 m/z at 50,000 resolution followed by 5 data depend (5 biggest peaks) ion trap MS/MS at 35 V normalize collision energy with an isolation window of 2 m/z. The data depend used dynamic exclusion to allow for obtaining MS/MS on more ions. The following parameters were used for the source: ESI voltage, 2 KV; capillary temperature, 200°C; capillary voltage, 40 V; and tube lens voltage, 150 V. Data analysis was performed using Thermo Proteome Discoverer 1.3. As identified proteins can be false hits or due to non-specific interactions, the analyses from all the biological replicates were compared to identify proteins that were found in more than one biological replicate. The list of proteins identified multiple times was then sorted based on the Proteome Discoverer Peptide Sorting Match (PSM) to rank hits.

### 2.6. Quantification of cellular metals

Quantification of cellular metals was performed as described previously (Duggal et al., 2020). Briefly, from overnight LB-agar plates grown at 30°C, *n* = 5 isolated colonies of WT and Δ*dgcO Pcc* were each inoculated into 3 mL of M9 minimal media [0.4% glucose (Research Products International) with 0.2% casamino acids (Becton, Dickinson and Co.), made using metal-free glassware (washed with 6 M HCl) and supplemented with various metals (Sigma-Aldrich) in 15 mL plastic conical tubes (VWR), then incubated overnight at 30°C with 225 rpm orbital shaking]. The list of metals and their concentrations can be found in Table 1. 15 mL of the same medium was inoculated with 100 μL of the overnight culture in 50 mL plastic conical tubes and grown to an OD_600_ ~ 0.8 at 30°C with 225 rpm orbital shaking. 10 mL of each culture was then transferred into pre-weighed 15 mL ICP-MS Teflon sample tubes and pelleted at 4000 × g for 10 min at 4°C. Supernatants were removed and the pellets weighed. Tubes were then flash frozen in liquid N_2_ and submitted for analysis by the Penn State Laboratory for Isotopes and Metals in the Environment (LIME) (University Park, PA, United States). Sample digestion and analysis was performed by LIME; briefly, bacterial pellets were digested using nitric acid, then analyzed using a Thermo Fisher Scientific Icap RQ ICP-MS instrument. Statistical significance was established using a 2-tailed Student’s *t*-test in Igor Pro 6.10 (Wavemetrics, 2009), metal content (in parts per billion) was normalized to cell pellet mass.

**Table 1:** Metals added to M9 media for quantification by ICP-MS.

Metal	Concentration in media
CaCl_2_•2H_2_O	4 μM
MnCl_2_•4H_2_O	2 μM
ZnSO_4_•7H_2_O	2 μM
CoCl_2_•6H_2_O	0.4 μM
CuCl_2_	0.4 μM
NiCl_2_	0.4 μM
NaMoO_4_•2H_2_O	0.4 μM
Na_2_SeO_3_	0.4 μM
H_3_BO_3_	0.4 μM
FeCl_3_	10 μM

### 2.7. Bacterial growth with additives

To assess growth in the presence of Cu, Mn and Zn, 1 M stocks of CuCl_2_, MnCl_2_•4H_2_O and ZnSO_4_•7H_2_O were made in H_2_O. These were then serially diluted in H_2_O to 0.001 M and added to M9 media (supplemented with 0.4% glucose and 0.2% casamino acids) to concentrations identical to those in ICP-MS experiment (Table 1). Additionally, the three 1 M stock solutions were diluted into LB to final working concentrations of 0 mM (LB with no additional metal), 3 mM, 9 mM, and 12 mM.

To assess carbon utilization, a 50% (v/v) stock solution of glycerol (Sigma-Aldrich) and a 25% (w/w) stock solution of arabinose (Acros Organics) were made in H_2_O. Appropriate volumes of these stock solutions were added to M9 media containing no carbon sources for final concentrations of 2 and 4% for glycerol, and 1 and 3% for arabinose.

For growth assays in the presence of Cu, Mn, Zn, and the sole carbon source utilization assays, *Pcc* WT and Δ*dgcO* were grown overnight on LB agar plates at 30°C. Four isolated colonies of each strain were inoculated into 3 mL LB broth in 15 mL plastic culture tubes (VWR), and grown overnight at 30°C with 225 rpm shaking. 196 μL of the appropriate media was added to wells of a 96 well plate, and the overnight cultures were diluted in fresh LB and were added to the wells to a normalized 0.005 OD_600_ starting concentration (~4 μL for each well).

For growth in the presence of FeCl_3_, *Pcc* WT and Δ*dgcO* were grown overnight on LB agar plates at 30°C. Three isolated colonies of each strain were inoculated into 3 mL LB broth in 15 mL plastic culture tubes (VWR), and grown overnight at 30°C with 225 rpm shaking. 980 μL of M9 media (supplemented with 0.4% glucose) with the appropriate concentration of FeCl_3_ (1 M stock solution made in H_2_O) was added to wells of a 24 well plate, and the overnight cultures were diluted in fresh LB to a normalized OD_600_ = 1.0, 20 μL of these normalized cultures was then added to the wells of the 24 well plate.

For all experiments, plates were covered by BreathEasy^™^ (Diversified Biocience) film and placed into a Biotek Epoch 2 plate reader for 24 h at 30°C with 228 cpm continuous orbital shaking, with OD_600_ being measured every 15 min. After the growth assay was completed, means and standard deviations for each strain were calculated, and the OD_600_ values were plotted as a function of time in Igor Pro 6.10.

### 2.8. Quantification of extracellular signaling molecules

Production of extracellular signaling molecules was measured using the methods described previously, with modifications (Surette et al., 1999). The AHLs 3-oxo-C6-HSL and 3-oxo-C8-HSL, were quantified, along with autoinducer-2 (AI-2). A list of control and reporter strains, alongside their role in these experiments, can be found in Table 2.

**Table 2:** List of *Vibrio* reporter and control strains used in the quantification of extracellular signaling molecules.

Strain	Genotype	Role	Source
*V. fischeri* JHK007	ES114 Δ*ainS*Δ*luxR*-*luxI*, P_luxI_-*lux*CDABEG	Nonluminescent, negative control for AHL production	(Colton et al., 2015)
*V. fischeri* DJ01	ES114Δ*ainS*Δ*luxR*-*luxI*, *luxR*^ES114^, P_luxI_-luxCDABEG	3-oxo-C6-HSL reporter	(Colton et al., 2015)
*V. fischeri* DC22	ES114Δ*ainS*Δ*luxR-luxI*, mutant *lux*R^B^ (MJ1-T33A, R67M, S116A, M135I), P_luxI_-*lux*CDABEG	3-oxo-C8-HSL reporter	(Colton et al., 2015)
*V. campbellii*	BB120 Wild-type	Positive control for AI-2, AHL production	(Colton et al., 2015)
*V. campbellii* MM32	luxN::Cm, luxS::Tn5Kan	AI-2 reporter	(Miller et al., 2004)
*V. campbellii* BB151	luxA::Tn5lac	Nonluminescent, negative control for AI-2 production	(Surette et al., 1999)

Briefly, cell free supernatants of WT and Δ*dgcO Pcc* were obtained by pelleting 1 mL cells grown in LB or M9 + Lettuce Leaf Lysate (LLL) media (3 mL inoculated with a 1:100 dilution of an overnight culture, grown at 30°C, 225 rpm orbital shaking) at the appropriate OD_600_ (OD_600_ = ~0.5, ~0.75, ~1.0, or ~ 1.2), followed by 0.22 μm filter sterilization of supernatant.

LLL was prepared as described previously (Burns et al., 2017). Briefly, 610.12 g of organic romaine lettuce hearts were washed then macerated using a manual juicer (Lexen). Lysate was then spun at 35,000 rpm (11pprox. 100,000 × g) for 45 min in a Beckman-Coulter Optima XE-90 ultracentrifuge. The supernatants were filter sterilized using 0.22 μm filters before use. LLL was stored at 4°C for sort-term, or at −80°C for long-term storage. M9 + LLL media was made by adding 12 μL LLL to 3 mL M9 media (0.4% glucose (Research Products International) with 0.2% casamino acids (Becton, Dickinson and Co.)) immediately before inoculation with bacteria (Burns et al., 2017).

Cell-free supernatants of the *V. campbellii* BB120 and BB151 control strains were prepared in a similar way to those of *Pcc*, but by streaking on LB-Salt (LBS) plates and growing bacteria in Autoinducer Bioassay (AB) (per liter: 17.5 g NaCl, 12.3 g MgSO_4_, 2.0 g Casamino acids, 10 mL 1 M pH 7.0 Potassium phosphate buffer, 10 mL 0.1 M L-arginine, 10 mL glycerol, all in H_2_O) medium to a 0.65 final OD_600_ (Surette et al., 1999).

All *Vibrio* reporter strains were grown overnight on LBS plates at 30°C, then a single colony was inoculated into 3 mL AB media at 30°C with 225 rpm shaking in 15 mL plastic culture tubes. These cultures were serially diluted to 1:5,000 with fresh AB, and used immediately after preparation.

To measure extracellular signaling molecule production, 40 μL 1:5,000 of the appropriate reporter culture was added to 160 μL cell-free supernatant of *Pcc* experimental or *V. campbellii* control strains in 96 well white/clear bottom plates (Thermo Scientific, 165306). For each biological replicate of *Pcc*, 2 technical replicates were used. Plates were covered using BreatheEasy film (Diversified Bioscience). Luminescence and OD_600_ were measured over 12 h using a Biotek Cytation5 plate reader 30°C with 180 cpm orbital shaking.

Signaling molecule production was quantified by comparing specific luminescence (LUM/OD_600_) values of wells containing the appropriate reporter strains grown in the presence of cell-free spent media from WT or Δ*dgcO Pcc* strains.

## 3. Results

### 3.1. WT vs. Δ*dgcO Pcc* exhibit differentially regulated transcripts when grown aerobically

**Figure 1:**
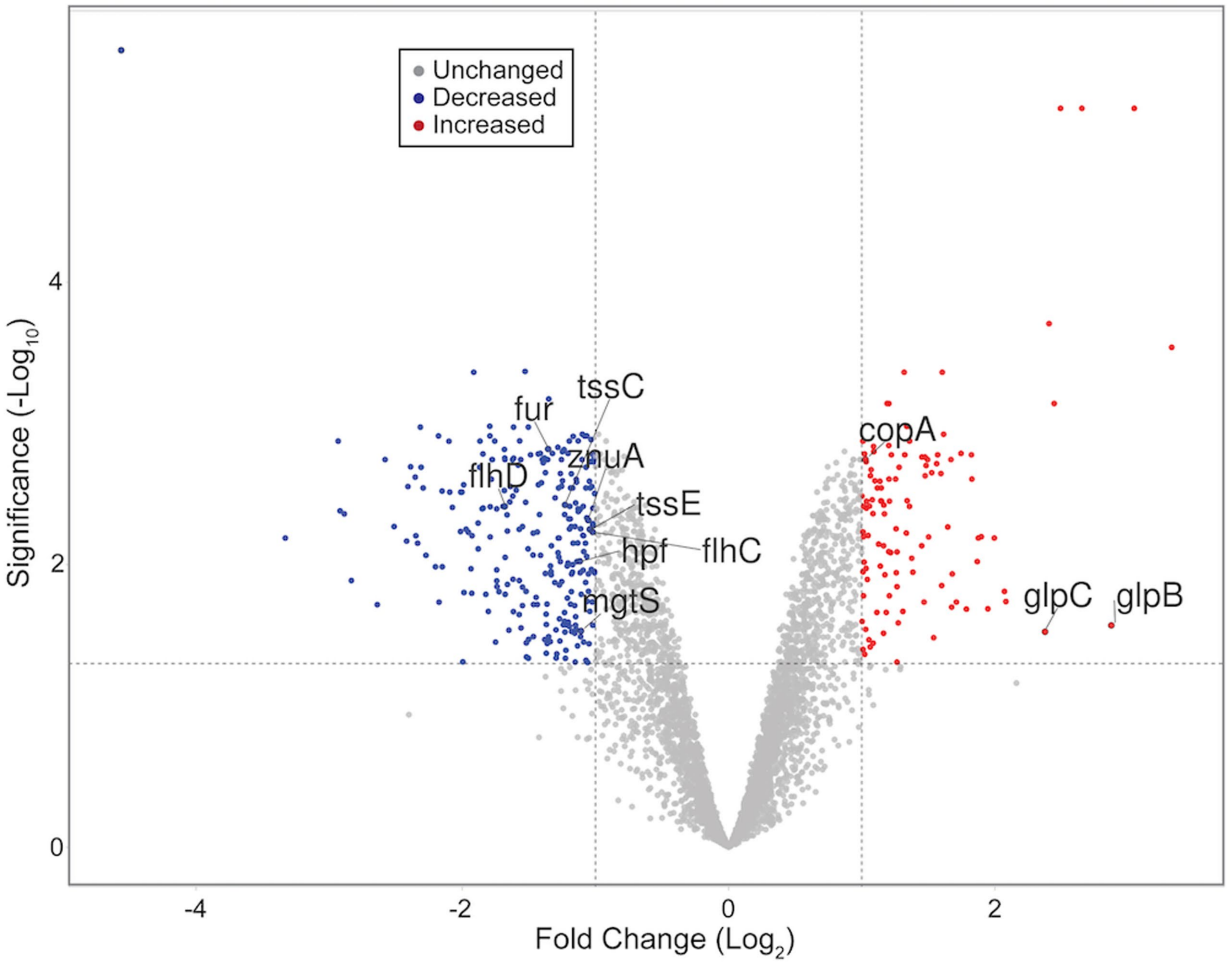
Volcano plot representing all quantified transcripts between WT and Δ*dgcO Pcc* grown aerobically. The horizontal dashed line signifies an FDR = 0.05 (−log_10_(0.05) = 1.3), while vertical dashed lines represent log_2_FC ± 1. Blue dots represent transcripts with significantly decreased, while red dots represent those with a significant increase in abundance in the Δ*dgcO* strain compared to WT. Representative transcripts labeled. Plot created using VolcaNoseR (Goedhart and Luijsterburg, 2020).

**Figure 2:**
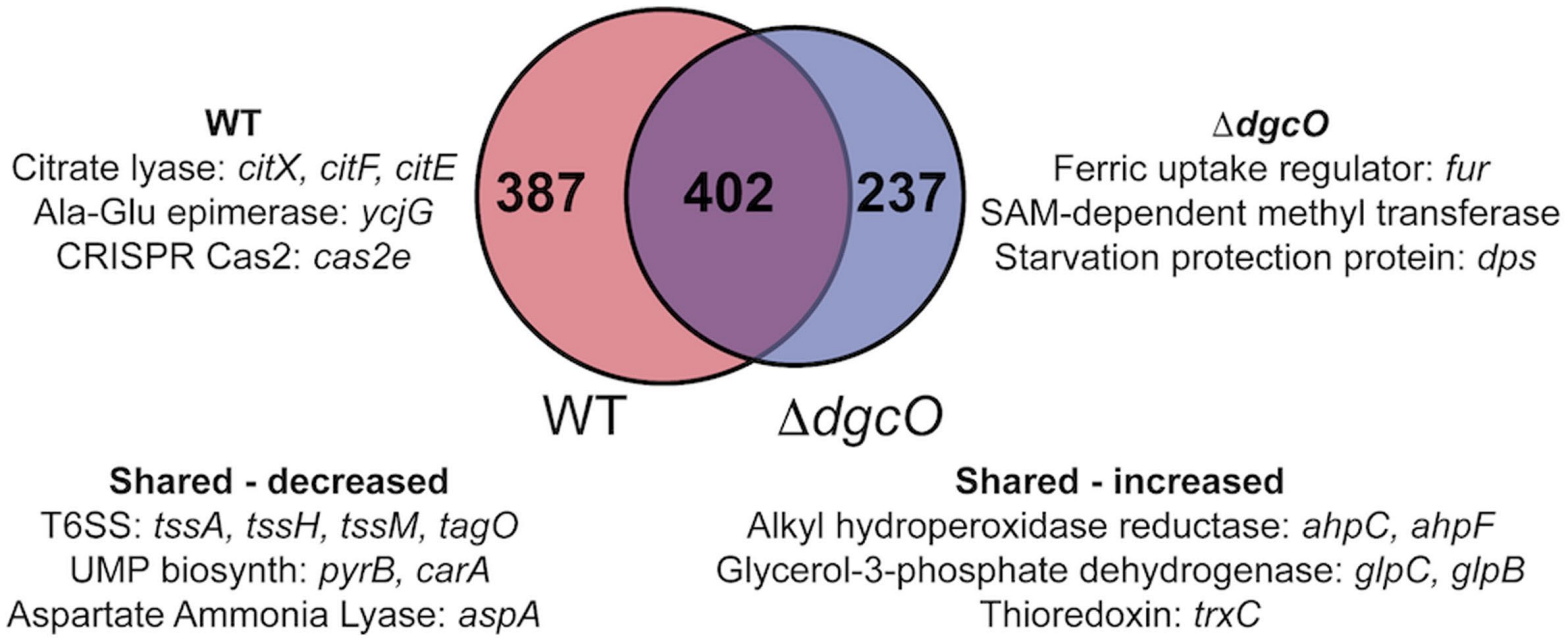
Venn diagram depicting overlap between transcripts that are differentially expressed in aerobic vs. anaerobic WT and aerobic vs. anaerobic Δ*dgcO.* Shared and unique genes with the largest differential expression are listed.

RNA sequencing and differential expression analysis revealed the differential expression of 372 transcripts between WT and Δ*dgcO Pcc* strains grown aerobically, but no significantly differentially expressed genes between WT and Δ*dgcO* grown anaerobically. The 372 aerobically differentially expressed transcripts correspond to approximately 9% of the genome (Figure 1). It is important to note that a large percentage of transcripts (330 transcripts, 88.7% of the total) have a subtle, 1 < |log_2_FC| < 2, change in their expression levels, with 237 transcripts (63.7% of total) having a change in expression of 1 < |log_2_FC| < 1.5. The majority (258, 69.4%) of differentially expressed transcripts had lower while 114 (30.6%) had higher expression levels in the Δ*dgcO* strain. A breakdown of the fold changes for each condition can be found in Table 3. The functions with the largest number of transcripts showing a decrease in abundance include the type 6 secretion system (T6SS) (15 transcripts), flagellar elements (8), virulence factors (5), and transcripts related to iron homeostasis and transport (4). Various transporters (20), methyl-accepting chemotaxis proteins (6), and transcripts related to carbohydrate metabolism (17) have increased transcript abundance in Δ*dgcO* compared to WT. Figure 2 shows a comparison between the numbers and identities of genes expressed between WT and Δ*dgcO.* A full list of quantified transcripts can be found in Supplementary Data Sheet 2, while a table classifying transcripts differentially expressed between WT and Δ*dgcO Pcc* grown aerobically can be found in Supplementary Table S1 (hypothetical proteins are excluded). All further Log_2_FC values refer to the differential expression of genes between WT and Δ*dgcO Pcc* grown aerobically, except where otherwise noted in Section 3.3.

**Table 3:** Number and percentage of differentially expressed transcripts between WT and Δ*dgcO Pcc* grown aerobically broken down based on level of differential expression.

Change	Lower expression in Δ*dgcO*	Higher expression in Δ*dgcO*
1 < |log_2_FC| < 1.5	156 (60.5%)	81 (71.1%)
1.5 ≤ |log_2_FC| < 2	71 (27.5%)	22 (19.3%)
2 ≤ |log_2_FC| < 3	29 (11.2%)	9 (7.9%)
3 ≤ |log_2_FC|	2 (0.8%)	2 (1.8%)

A total of 372 transcripts were differentially expressed, 258 had lower, 114 had higher expression levels in Δ*dgcO Pcc* compared to WT.

### 3.2. Quantification of c-di-GMP

**Figure 3:**
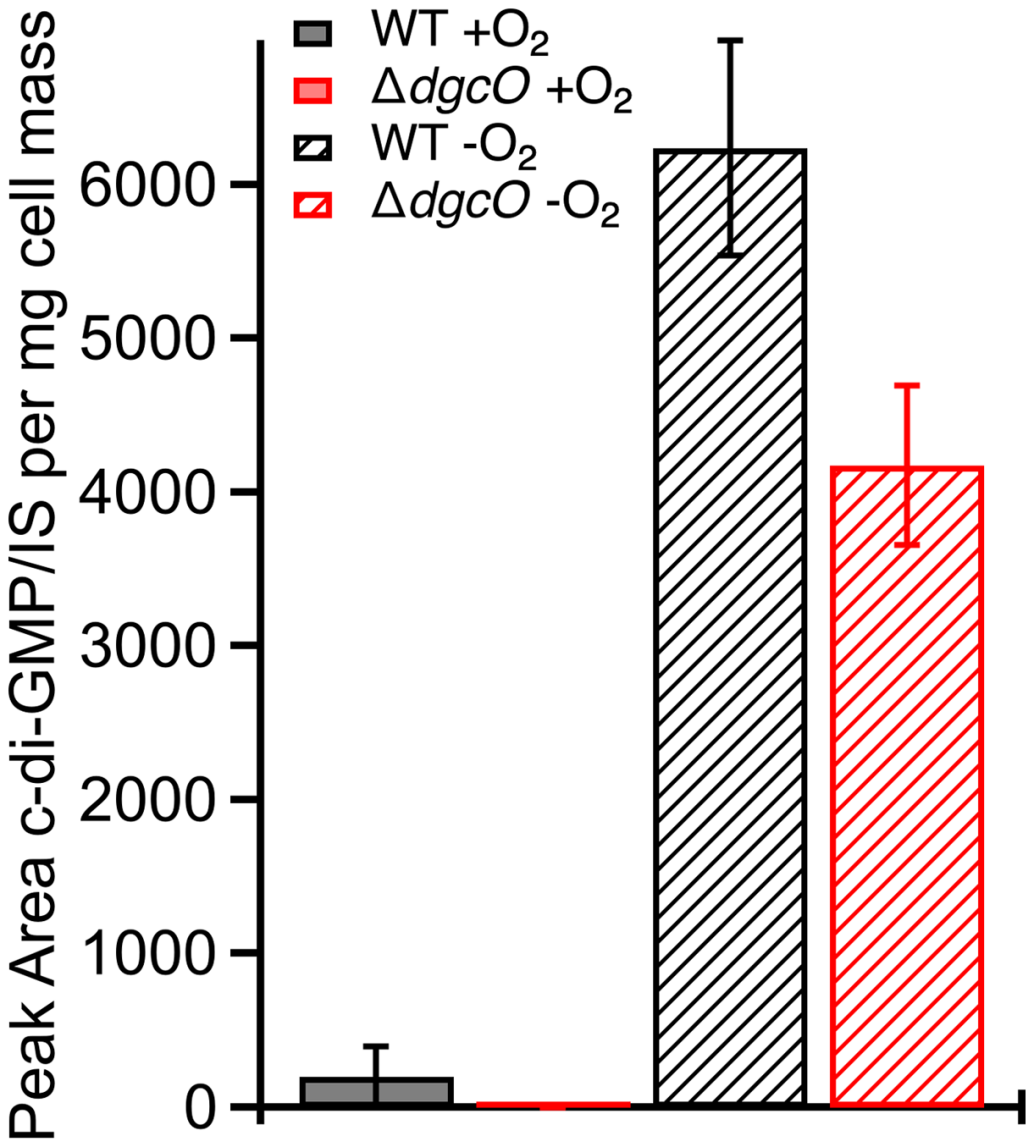
Levels of c-di-GMP quantified from WT and Δ*dgcO Pcc* grown aerobically or anaerobically. No significant difference between c-di-GMP levels was found between the two strains grown aerobically, but when grown anaerobically, both strains show a significant increase compared to aerobic growths. Additionally, the Δ*dgcO* strain has a significantly lower concentration compared to WT when grown anaerobically.

The subtle transcript level changes between WT and Δ*dgcO Pcc* suggest that most regulation by DgcO happens through localized c-di-GMP signaling and not through major changes in the cellular c-di-GMP pool. To test this hypothesis, c-di-GMP was quantified from WT and Δ*dgcO Pcc* grown under aerobic and anaerobic conditions. Aerobically, the levels of c-di-GMP were found to be low for both strains, with c-di-GMP concentration in the Δ*dgcO* strain being under the limit of quantification. Overall, aerobic c-di-GMP concentrations show no significant difference between WT and Δ*dgcO Pcc* (Figure 3). When comparing c-di-GMP levels between WT and Δ*dgcO Pcc* grown anaerobically, the WT strain was found to have a higher c-di-GMP concentration compared to the Δ*dgcO* strain (Figure 3). When comparing c-di-GMP levels of the same strain between the two conditions, anaerobically grown WT and Δ*dgcO Pcc* both had a significant increase in their c-di-GMP levels compared to cells of the same strain grown aerobically (Figure 3).

### 3.3. Δ*dgcO* Strain exhibits altered effects on transcripts in response to anaerobic conditions

The differences between *Pcc* WT transcript levels for cells grown aerobically vs. anaerobically were found to vary when compared to those identified in a comparison of *Pcc* Δ*dgcO* grown aerobically vs. anaerobically. Differences were found in the number of transcripts with altered levels (789 vs. 639, respectively), gene identities, and Log_2_FC’s when comparing the WT and Δ*dgcO* strains. For example, there are no differences in transcript levels for genes encoding ribosomal proteins or elongation factors in *Pcc* WT, but *Pcc* Δ*dgcO* exhibits 1–1.5 Log_2_ fold changes in 18 transcripts encoding ribosomal proteins and elongation factors. Of the 639 altered transcripts identified in *Pcc* Δ*dgcO* grown under anaerobic vs. aerobic conditions, 89 are also altered when comparing aerobically grown *Pcc* Δ*dgcO* vs. WT strains. In all cases, transcript levels that have higher expression levels in aerobic *Pcc* Δ*dgcO* vs. WT also have higher expression levels in *Pcc* Δ*dgcO* aerobic vs. anaerobic datasets. The remaining 550 transcripts that were identified in the *Pcc* Δ*dgcO* aerobic vs. anaerobic comparison suggest that *Pcc* does not transition properly between aerobic and anaerobic environments in the absence of DgcO. All of the 50 most differentially expressed transcripts identified when comparing *Pcc* Δ*dgcO* grown aerobically vs. anaerobically were also identified in the comparison of *Pcc* WT aerobic vs. anaerobic RNAseq. Of this set of transcripts, 75 were found to share either increase or decrease in transcript levels between *Pcc* WT and Δ*dgcO*, although the magnitude of the Log_2_FC between aerobically and anaerobically grown cells varied (Supplementary Table S2). The remaining 25 transcripts exhibited opposing changes in transcript levels, with the 16 transcripts with the largest decrease in abundance in *Pcc* Δ*dgcO* all showing increased expression in *Pcc* WT. While six of the genes encode for putative or hypothetical proteins, genes encoding parts of the type VI secretion system (HER17_04885, *tssM*, *tssH*, *tssA*) and a sigma-54-dependent Fis family transcriptional regulator (HER17_04895), which may be involved in regulation of virulence, have decreased levels in *Pcc* Δ*dgcO* but have increased abundance in *Pcc* WT. These differences may be involved in the previously observed decrease in virulence factor excretion and virulence in a potato host by *Pcc* Δ*dgcO*, as compared to *Pcc* WT (Burns et al., 2017).

**Figure 4:**
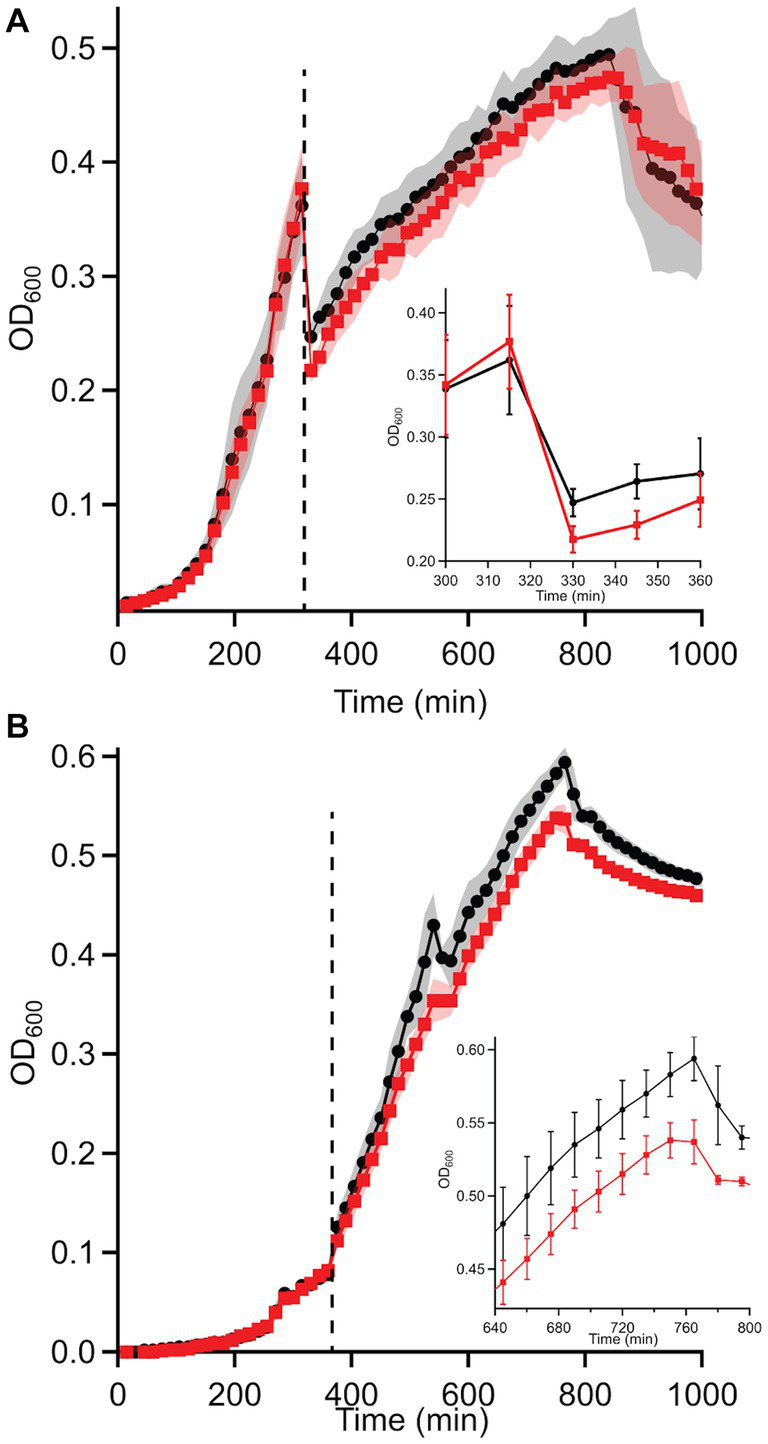
Growth curves of WT and Δ*dgcO Pcc* grown in M9 + LLL media transitioning from **(A)** aerobic to anaerobic and **(B)** anaerobic to aerobic conditions (*n* = 8). Dashed vertical line indicated moving cells from aerobic to anaerobic environment (after 315 min). The inset on panel A shows the transition from aerobic to anaerobic conditions, with a higher drop in turbidity observed in the Δ*dgcO* strain between 330 and 360 min of growth (the first 30 min of aerobic growth). On panel **(B)**, the inset shows the region where both strains reached maximum OD_600_.

Based on these discrepancies, the transition of WT and Δ*dgcO Pcc* from aerobic to anaerobic growth was studied. Results showed that while there is no difference between adaptation to anaerobic or aerobic conditions in LB (Supplementary Figures S1A,B), when grown in M9 + LLL media, the Δ*dgcO* strain exhibits a longer lag when moved into the anaerobic chamber compared to the WT strain; after 30 min, the two strains grow identically again (Figure 4A). When moved from anaerobic to aerobic conditions, the WT strain exhibited a higher final OD_600_ than the Δ*dgcO* strain (Figure 4B).

### 3.4. DgcO affects transcript levels of flagellar genes and interacts with chemotaxis proteins

**Figure 5:**
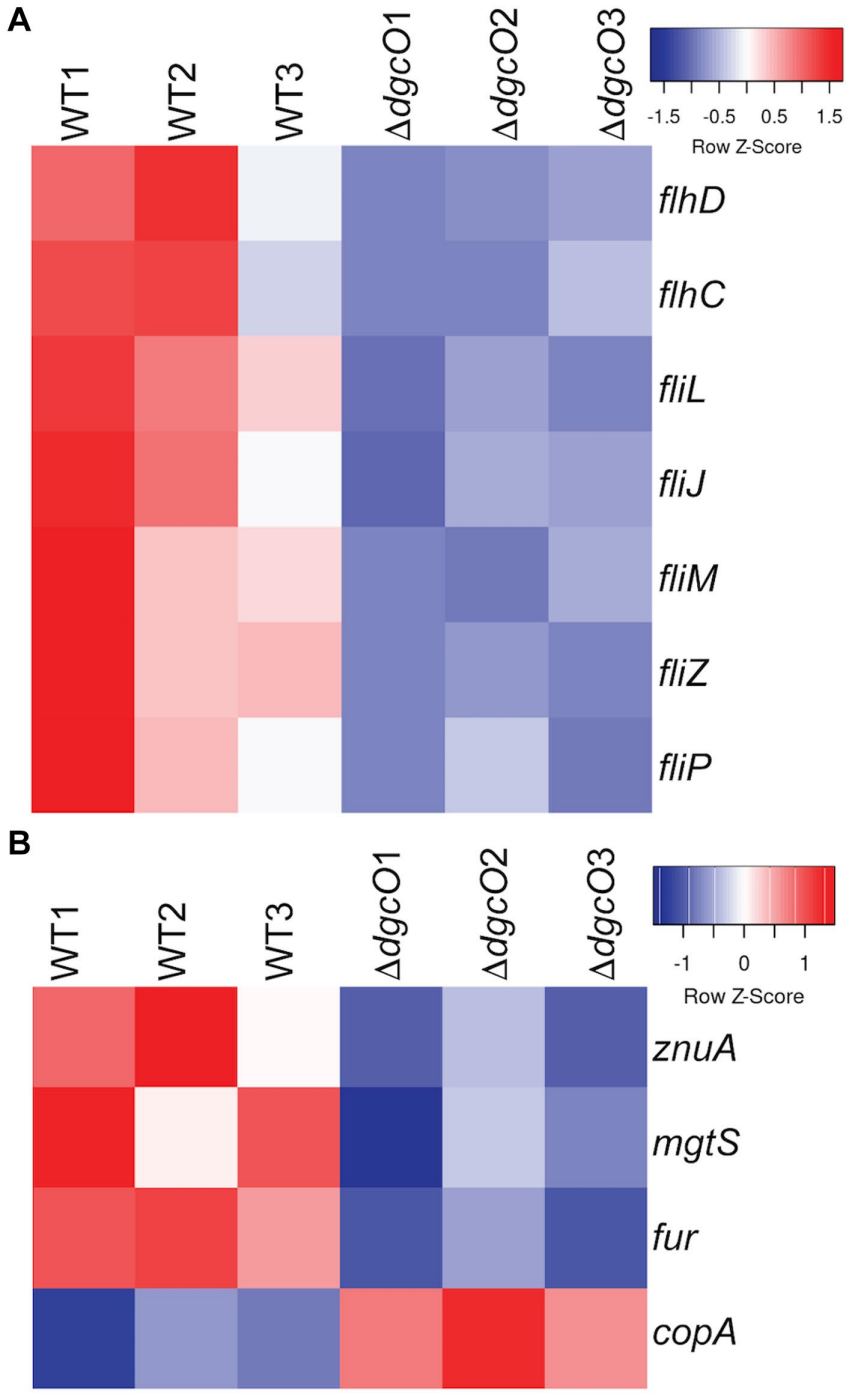
Heatmaps of expression levels of transcripts in WT and Δ*dgcO Pcc* related to flagellar **(A)** and metal homeostasis **(B)** functions. Transcript levels of flagellar structural elements and the master flagellar regulator *flhDC* were found to be decreased upon *dgcO* deletion. Additionally, transcript levels of the zinc importer *znuA*, the small protein *mgtS*, and the ferric uptake regulator *fur* were decreased, while the copper exporter *copA* showed increased expression upon *dgcO* deletion.

Transcriptomic results revealed the decrease in transcript levels of the *fliJ*, *fliL*, *fliM*, *fliP* flagellar structural and motor proteins (avg. ~ −1.16 Log_2_FC), and the *fliZ* (~ −1.90 Log_2_FC) and *flhC* (~ −1.02 Log_2_FC), *flhD* (~ −1.68 Log_2_FC) transcriptional regulators in *Pcc* Δ*dgcO*, as compared to WT (Figure 5A). The transcription factors *flhC* and *flhD* form the *flhDC* complex *in vivo*; this complex is known to positively regulate motility in *P. carotovorum* (Chatterjee et al., 2009). Based on these findings, and previously published interaction data suggesting that DgcO interacts with the chemotaxis proteins TarH, CheA, and CheW, Ni-NTA was used to pulldown His-tagged DgcO and interacting proteins (Burns et al., 2017). The pull-down was performed with cleared lysate, so it is possible that DgcO makes additional interactions with membrane proteins that were not identified in these experiments. Results indicate that DgcO interacts with the chemotaxis protein CheY, which upon phosphorylation serves as a switch signal to the flagellar motor, suggesting that DgcO localizes with the motility machinery complex. High confidence hits (as assessed by #PSM) for peptides eluting in the same fraction as DgcO can be found in Table 4; it is important to note that many peptides are found as low-quality hits, resulting in a large number of potential interacting partners. Full list of peptide hits from the pulldown can be found in Supplementary Data Sheet 3.

**Table 4:** Proteins with the highest quality hits (as assessed by #PSM) interacting with DgcO as identified in Ni-NTA pulldown.

Protein	Function
TsaA	Peroxidase
LuxS	S-ribosylhomocysteine lyase
Mdh	Malate dehydrogenase (NAD)
Tsf	Translation elongation factor Ts (EF-Ts)
OsmY	Periplasmic protein
SdhA	Succinate dehydrogenase subunit A
FusA	Translation elongation factor 2 (EF-2/EF-G)
NuoG	NADH dehydrogenase subunit G
KatG	Catalase-peroxidase
CheY	Chemotaxis regulatory protein CheY
ArnA	Bifunctional UDP-glucuronic acid decarboxylase/UDP-4-amino-4-deoxy-L-arabinose formyltransferase
SlyD	FKBP-type peptidyl prolyl cis-trans isomerase/Apo-metallochaperone SlyD
Crp	cAMP-regulatory protein
Fur	Ferric uptake regulator

It is important to note that ArnA, SlyD, Crp, and Fur are known common contaminants in Ni-NTA pulldowns (Bolanos-Garcia and Davies, 2006; Chen et al., 2017). Arabinose utilization was tested but yielded no significant differences between the strains (Supplementary Figure S1E).

### 3.5. DgcO alters metal homeostasis

**Figure 6:**
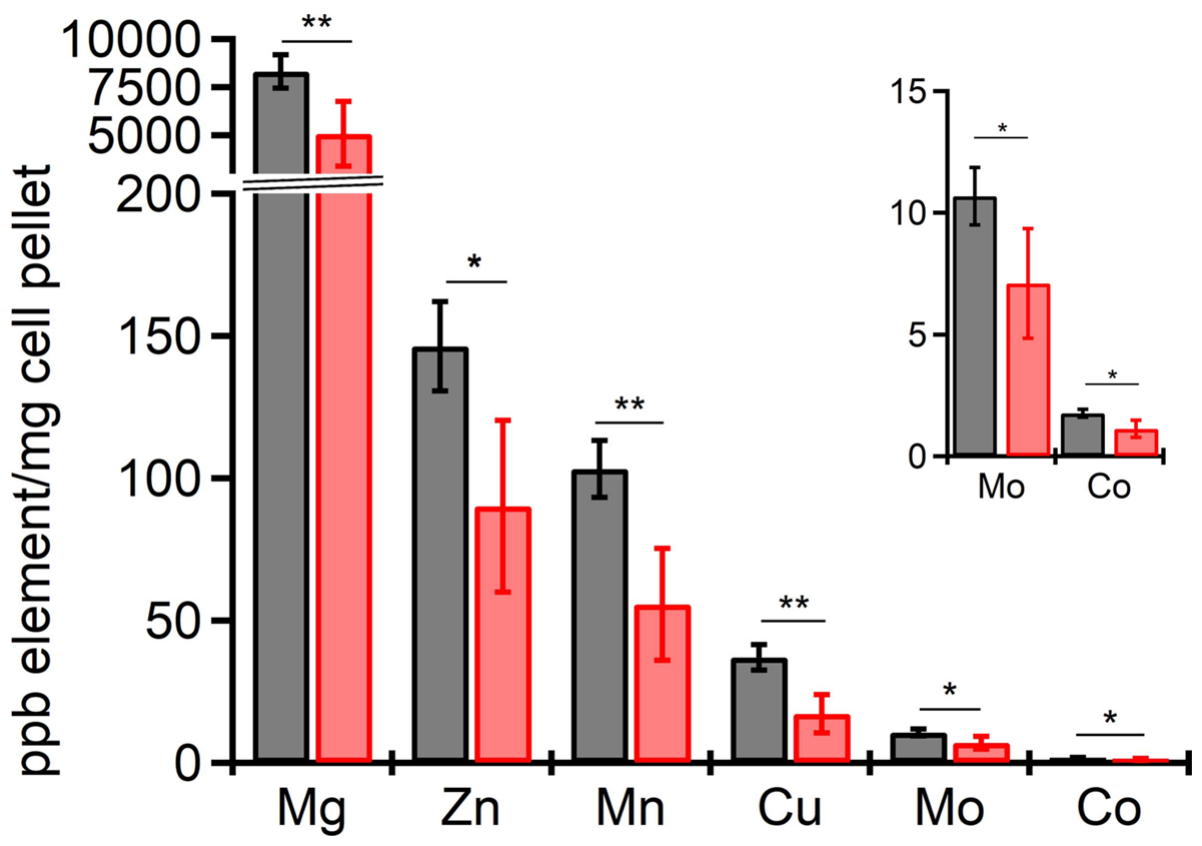
Cellular levels physiologically relevant metals quantified by ICP-MS in aerobically grown WT and Δ*dgcO Pcc*. Six metals, Cu, Mg, Mn, Co, Mo, and Zn have been found to be contained in significantly lower levels in Δ*dgcO* compared to WT (* denotes *p* < 0.05, ** denotes *p* < 0.01, as found using Student’s *t*-tests, WT *n* = 4, Δ*dgcO n* = 5).

From the RNAseq data, the ferric uptake regulator transcription factor, *fur* (~ −1.36 Log_2_FC), and various metal transporters were found to be differentially expressed in aerobically grown Δ*dgcO*, as compared to WT (Troxell and Hassan, 2013). Genes annotated as responsible for transporting iron (avg. ~ 1.25 Log_2_FC) and the *copA* copper (I) transporter (~ 1.03 Log_2_FC) have increased transcript levels in *Pcc* Δ*dgcO*, while the zinc ABC transporter substrate-binding protein *znuA* (~ −1.05 Log_2_FC) and the magnesium starvation response regulator *mgtS* (~ −1.11 Log_2_FC) had decreased transcript levels in *Pcc* Δ*dgcO* (Figure 5B). To determine if the differential expression of genes for metal transporters, metal homeostasis, and transition metal-containing cofactor synthesis affected cellular metal levels, ICP-MS was used to quantify cellular metal content in WT and Δ*dgcO Pcc* grown in minimal media (to allow for complete control of metal concentrations). Out of the 10 metals tested, six showed statistically significant differences in levels, with WT containing higher levels of each metal (Figure 6). The six metals with differences in their concentrations, Co, Cu, Mo, Mg, Mn, and Zn all play an important role in the cell, often serving as cofactors for enzymatic activity. Interestingly, iron levels were not found to be different between the two strains, suggesting possible compensatory effects to the decreased *fur* transcript levels. No difference was found when cells were grown in M9 in the presence of various concentrations of FeCl_3_ (Supplementary Figure S1C). Similarly, when cells were grown in M9 media supplemented with Cu, Mn, and Zn at concentrations seen in ICP-MS, no difference was found between WT and Δ*dgcO Pcc* (Supplementary Figure S1D).

**Figure 7:**
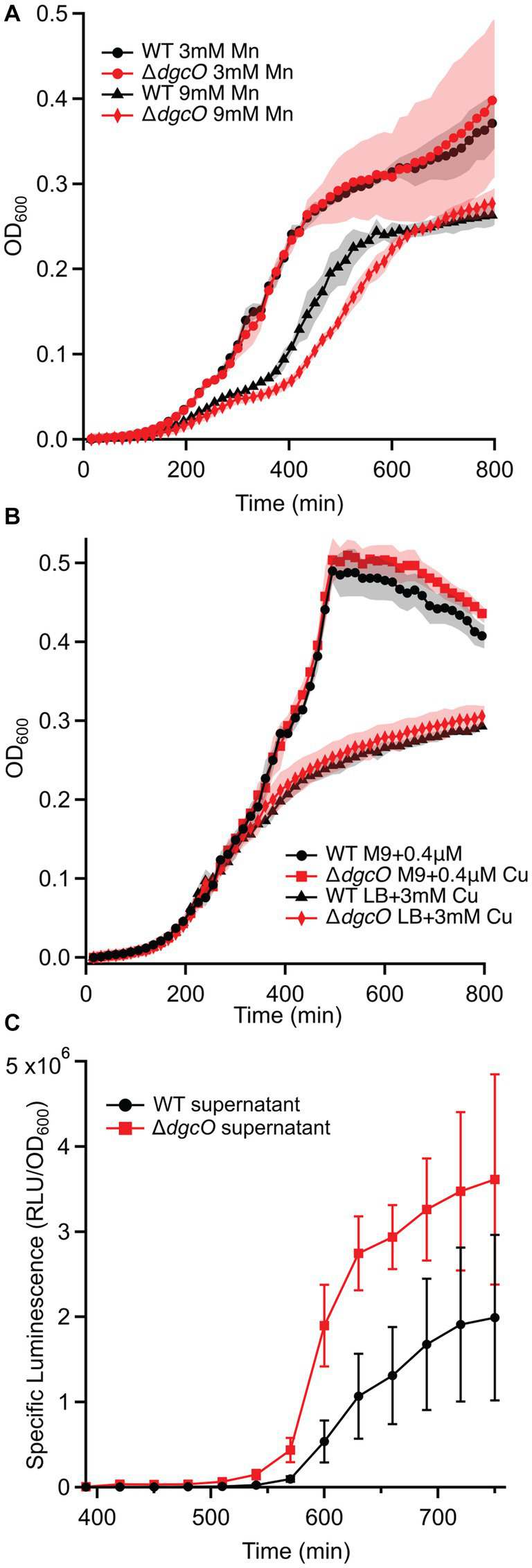
Growth curves of WT and Δ*dgcO Pcc* grown in LB supplemented with 3 mM and 9 mM Mn (*n* = 4) **(A)**. Strains grown in M9 + glucose or LB with varying concentrations of CuSO_4_ **(B)**. Specific luminescence of *V. campbellii* MM32 cells when grown in the presence of WT and Δ*dgcO Pcc* supernatant harvested from cells grown to OD_600_ = 0.50 in LB (*n* = 3) **(C)**.

Growth assays performed in LB supplemented with Cu, Mn, and Zn also showed no difference in copper tolerance between the strains (Figure 7B), while neither strain could tolerate the high levels of zinc. With manganese, no difference was seen at 3 mM and 12 mM concentrations, but at 9 mM, the Δ*dgcO* strain showed an increased time (approx. 45 min) to mid-log, but reached the same stationary phase OD_600_ as WT (Figure 7A).

### 3.6. PCWDE expression is reduced in Δ*dgcO*

It was previously demonstrated that the deletion of *dgcO* reduces PCWDE production by ~15% under aerobic conditions, but the regulation of this activity was unknown (Burns et al., 2017). Transcriptomic data shows that pectate lyase (avg. ~ −1.29 Log_2_FC), polysaccharide lyase (~ −1.04 Log_2_FC), and cellulase (~ −1.19 Log_2_FC) transcript levels show a decrease in Δ*dgcO Pcc*, providing evidence that *Pcc*DgcO controls PCWDE expression.

### 3.7. DgcO plays a role in regulating T3SS and T6SS expression

The expression of the type 3 secretion system (T3SS) and T6SS was shown to be regulated by c-di-GMP in *Pseudomonas aeruginosa*, with T3SS being expressed at low, and T6SS being expressed at high c-di-GMP levels, respectively (Dadashi et al., 2021). The exact mechanism by which the expression of these systems is regulated by c-di-GMP is not fully understood and previously has not been studied in *Pcc.* Upon *dgcO* deletion (and thus the removal of c-di-GMP synthesized by DgcO) differential expression of genes involved in T3SS and T6SS formation and activity in *Pcc* follows the pattern described above. A key T3SS ATPase, *sctN*, shows increased expression in Δ*dgcO* (~ 1.04 Log_2_FC). In *Chlamydia trachomatis*, when active, SctN, plays a role in unfolding the chaperone-effector complex, a step necessary for effector secretion and host colonization (Grishin et al., 2018). Besides *sctN*, the decrease in transcript levels of the *flhDC* regulatory complex also suggests transcriptional control of T3SS, as *flhDC* is a known regulator of T3SS expression in *P. aeruginosa* (Soscia et al., 2007). Another possible T3SS regulator is the sigma factor RpoE, which plays a role in response to cellular stress, and has been found to have a decrease of ~ −1.07 Log_2_FC in its transcript levels (Flores-Kim and Darwin, 2012).

Deletion of *Pcc* DgcO results in the decrease in expression of *tssB*, *tssC*, *tssE*, *tssG*, and *tssJ* (avg. ~ −1.19 Log_2_FC); these genes all encode for T6SS structural elements. Additionally, a total of 9 transcripts annotated as Hcp family effectors also showed and average decrease in transcript levels of ~ −1.88 Log_2_FC. These results suggest c-di-GMP-dependent control of both the assembly of T6SS and the synthesis of effectors used in interspecies competition.

### 3.8. Regulation of cell-cell communication by DgcO

Besides T6SS, systems involved in interactions with other microbes appear to be regulated by DgcO. The quorum sensing master regulator, *expR*, is shows a transcript abundance decrease ~ −1.38 Log_2_FC, along with additional LuxR family transcriptional regulators (avg. ~ −1.48 Log_2_FC). These results, taken together with previously quantified AHL levels, suggest that the deletion of *dgcO* causes no defect in AHL synthesis, but it is possible that AHL gets exported from the cell through pumps, although no differential expression of known AHL-binding pumps was identified in the transcriptomic data (Burns et al., 2017).

In addition to AHL-mediated cell–cell communication, DgcO appears to have an effect on interspecies communication through localized signaling, as Ni-NTA pulldown showed that LuxS, a major component of the AI-2 synthesis pathway, associates with DgcO (Põllumaa et al., 2012; Joshi et al., 2016). A *Vibrio* reporter assay using the *V. campbellii* MM32 AI-2 reporter strain showed that when the reporters were grown in the presence of supernatant harvested from WT and Δ*dgcO Pcc* cells grown to OD_600_ = 0.50 in LB, specific luminescence of the reporters was higher when they were grown in the presence of supernatant from Δ*dgcO Pcc* (Figure 7C). Interestingly, when sterile-filtered supernatant was used from *Pcc* WT or Δ*dgcO* grown media to mimic a plant host (M9 + LLL), *V. campbellii* and *V. fischeri* reporters failed to grow, suggesting that lettuce extract induced *Pcc* excretion of inhibitory factors in a *dgcO*-independent manner.

### 3.9. DgcO affects translation-related genes

Transcripts encoding for a total of 6 ribosomal proteins (*rpmC*, *rpmI*, *rpmJ*, *rpsJ*, *rpsM*, and *rmsQ*) were found to have lower levels in *Pcc* Δ*dgcO* (avg. ~ −1.15 Log_2_FC), while the ribosome hibernation promoting factor *hpf* has an increase of ~1.12 Log_2_FC in transcript levels, as compared to WT (Ueta et al., 2008; Polikanov et al., 2012). Additionally, Ni-NTA pulldown identified the translation elongation factors FusA and Tsf to be interacting with *Pcc* DgcO, suggesting that DgcO plays a role in regulating translation.

### 3.10. Regulation of anaerobic processes

As mentioned, *Pcc* DgcO serves as an O_2_ sensor and diguanylate cyclase activity is greater aerobically. Based on transcriptomic data however, deletion of *Pcc* DgcO also modulates anaerobic processes, namely anaerobic citrate and glycerol metabolism, even under aerobic growth conditions. Genes encoding for citrate lyase and various associated proteins (*citCDEF* and *citX*) were had an average increase of ~1.80 Log_2_FC in their transcript levels, while two subunits of the anaerobic glycerol-3-phosphate dehydrogenase (*glpBC*) had an average increase of ~2.63 Log_2_FC in Δ*dgcO Pcc*, suggesting a possible c-di-GMP repression of these processes aerobically. However, when WT and Δ*dgcO Pcc* cells were grown aerobically with 2% or 4% glycerol as a sole carbon source, no significant difference was found between the two strains (Supplementary Figure S1F).

### 3.11. Putative retron identified in the *Pcc* genome

Based on RNA mapping and expression levels, a putative noncoding RNA has been identified upstream of a genomically encoded reverse transcriptase. This, along with the presence of an ATPase and a HNH effector immediately downstream of the reverse transcriptase, suggests that a possible retron is present in *Pcc* (Millman et al., 2020). Furthermore, expression of these three genes is decreased (~ −1.80 Log_2_FC) in the Δ*dgcO* strain, suggesting that DgcO might play a role in the regulation of this putative retron, which are believed to be at least partially controlled by cyclic nucleotides (Maxwell, 2021; Tal et al., 2021). However, so far c-di-GMP-dependent regulation of retrons has not been identified. While the architecture matches those previously reported (Millman et al., 2020), further validation is needed to confirm the presence and identity of the retron.

## 4. Discussion

Transcriptomic studies comparing aerobic and anaerobic growth of *Pcc* WT and Δ*dgcO* found that while the WT strain had more differentially expressed genes (789 vs. 639), a large portion (402 genes) of these genes were differentially expressed in both strains. When compared to differential expression data from *P. atrosepticum* grown under aerobic or anaerobic conditions, a number of functions show similar regulation between the two conditions, such as genes related to the TCA cycle (e.g., *sdh*CD), stress response (e.g., *trxC, ahpC*), or transporters (e.g., *copA*, PTS sugar transporters) (Babujee et al., 2012). Interestingly, a large number of genes (142) found to be differentially expressed between the same strains aerobically vs. anaerobically was also found to be differentially expressed between WT and Δ*dgcO* aerobically. These genes include the *citXDEF* operon and genes involved in T6SS expression. These results, along with the changes in growth exhibited by Δ*dgcO Pcc* when transitioning between aerobic and anaerobic conditions in M9 + LLL media (Figures 4A,B), suggest that DgcO plays an important regulatory role in *Pcc* transitioning between aerobic and anaerobic conditions.

When comparing WT and Δ*dgcO Pcc*, analysis identified transcript differential expression upon *dgcO* deletion only when cells are grown aerobically, which is supported by previous studies that DgcO diguanylate cyclase activity is highest in the presence of O_2_ (Burns et al., 2014). The results also identified changes in transcript levels relating to previously identified phenotypes, such as PCWDE production, AHL synthesis, biofilm formation, and motility (Burns et al., 2017). Possible explanations include control through riboswitches, direct binding of c-di-GMP (e.g., the cellulose biosynthesis protein BcsE, which contains a GIL domain), or through currently unidentified transcription factors (Weinberg et al., 2007; Xin et al., 2014). The widely studied c-di-GMP binding PilZ domain is not found in *Pcc*, suggesting the presence of additional, currently unidentified c-di-GMP domains (Ryjenkov et al., 2006).

LC-MS/MS quantification of c-di-GMP showed no significant difference in concentration between aerobically grown WT and Δ*dgcO*, further supporting the hypothesis that aerobic DgcO-dependent c-di-GMP signaling is primarily regulated through local concentrations rather than changes in the concentration of the cellular c-di-GMP pool. Interestingly, anaerobically, the Δ*dgcO* strain was found to have a significantly lower c-di-GMP concentration compared to WT, potentially loss of localized signaling that alters activity of other c-di-GMP metabolic enzymes, as previous work has demonstrated that DgcO is most active when oxygen is bound (Burns et al., 2014).

Transcriptomic data shows evidence of *dgcO* affecting the transcript levels of numerous transcription factors, such as *fur*, *flhDC*, and 19*xpr* (Põllumaa et al., 2012; Tanui et al., 2017), and genes known to be part of the transcription factors regulons also are differentially expressed, suggesting that *dgcO* may regulate cellular function by regulating transcription factors expression.

The decreased cellular metal content in Δ*dgcO* (Figure 6), and changes in Mn tolerance (Figure 7) are likely due to differential expression of *fur* and various metal pumps (Supplementary Table S1). In addition, the changes in metal concentration possibly affect previously observed decreased *Pcc* Δ*dgcO* biofilm formation, as Mg, Cu, Mn, and Zn are known to induce and stabilize biofilms in *Pectobacterium brasilense*, and in *Pcc* str. PC1 (Manjurul Haque et al., 2012; Burns et al., 2017; Haque et al., 2017). While a few proteins related to metal regulation were identified as interacting with DgcO, the hit probabilities were low and the proteins were only identified in a subset of the biological replicates, suggesting that metal levels are modulate by DgcO primarily by regulating transcription (Table 5). Decrease in metal content and related growth phenotypes of the Δ*dgcO* strain provide the first evidence of a GCS protein regulating cellular metal levels, although this putative regulation requires further investigation.

**Table 5:** Comparison of identified *Pcc*DgcO interacting proteins and transcripts from key cellular pathways.

	Interacting proteins	Transcripts
Quorum sensing	**LuxS**	LuxR family txn regulator (3)
Motility	**FliC**, FliD, CheY, CheZ	*fliM*, *fliJ, flip, fliL, flhC, flhD, fliZ, fliM*, methyl accepting chemotaxis protein (4)
ROS detoxification	**TsaA**,**Tpx**,**AhpF**, KatG, SodA	*ahpF*
Metal regulation	AfuA, Bfr, CysG1, Zur	*fur, ftnA, copA, znuA,* Fe permease (3), Fe transporter, Fe transporter/permease
Glycerol utilization	**GapA**,**GcvP**,**GpmA**,**GlpD**, Pkg	*glpB, glpC, ugpC,* glycerate kinase, *gapA*

Interacting proteins with the highest quality hits (as assessed by #PSM) are highlighted in bold.

Interactions of *Pcc*DgcO protein with the chemotaxis proteins CheW and CheY suggest regulation of these proteins through specific localized c-di-GMP signaling. Interestingly, in general, decreased c-di-GMP levels correlate with increased motility, but Δ*dgcO* was shown to have reduced motility compared to WT *Pcc* (Burns et al., 2017; Jenal et al., 2017). Additional chemotaxis and motility related proteins were identified in the pull-down experiments, as were a number of differentially expressed transcripts (Table 5). These data suggest that DgcO might possibly regulate motility through stabilizing the Che chemotaxis scaffold, producing c-di-GMP that binds to chemotaxis machinery, and modulating expression of motility and chemotaxis genes.

Overall, while these results only show low and moderate level changes in transcript levels, a model can be hypothesized by which DgcO primarily controls function through specific local c-di-GMP signaling, without a significant contribution to the overall cellular c-di-GMP pool. While this model requires further validation, it provides new insights into the cellular effects of oxygen-dependent c-di-GMP regulation in this important phytopathogen.

## Data availability statement

The datasets presented in this study can be found in online repositories. The names of the repository/repositories and accession number(s) can be found below: NCBI GEO - GSE214075.

## Author contributions

FF, NM, XL, and EW performed experiments and analyzed data. FF, NM, and EW wrote and edited manuscript. All authors contributed to the article and approved the submitted version.

## Funding

This work was supported by NSF CHE1352040 (EW), NSF CHE2003350 (EW), and Frasch Foundation Grant 824-H17 (EW).

## Conflict of interest

The authors declare that the research was conducted in the absence of any commercial or financial relationships that could be construed as a potential conflict of interest.

## Publisher’s note

All claims expressed in this article are solely those of the authors and do not necessarily represent those of their affiliated organizations, or those of the publisher, the editors and the reviewers. Any product that may be evaluated in this article, or claim that may be made by its manufacturer, is not guaranteed or endorsed by the publisher.
