# Supplementary material for: An O2-sensing diguanylate cyclase broadly affects the aerobic transcriptome in the phytopathogen Pectobacterium carotovorum
Source: Front Microbiol. 2023 Jul 7;14:1134742. doi: 10.3389/fmicb.2023.1134742 (PMC10360401; doi:10.3389/fmicb.2023.1134742)
Supplement: Supplementary file 1 [file Data_Sheet_1.PDF]

Supplementary Material

An O_2-Sensing Diguanylate Cyclase Broadly Affects the Aerobic Transcriptome in the Phytopathogen *Pectobacterium carotovorum*

Florian J. Fekete, Nick J. Marotta, Xuanyu Liu, Emily E. Weinert

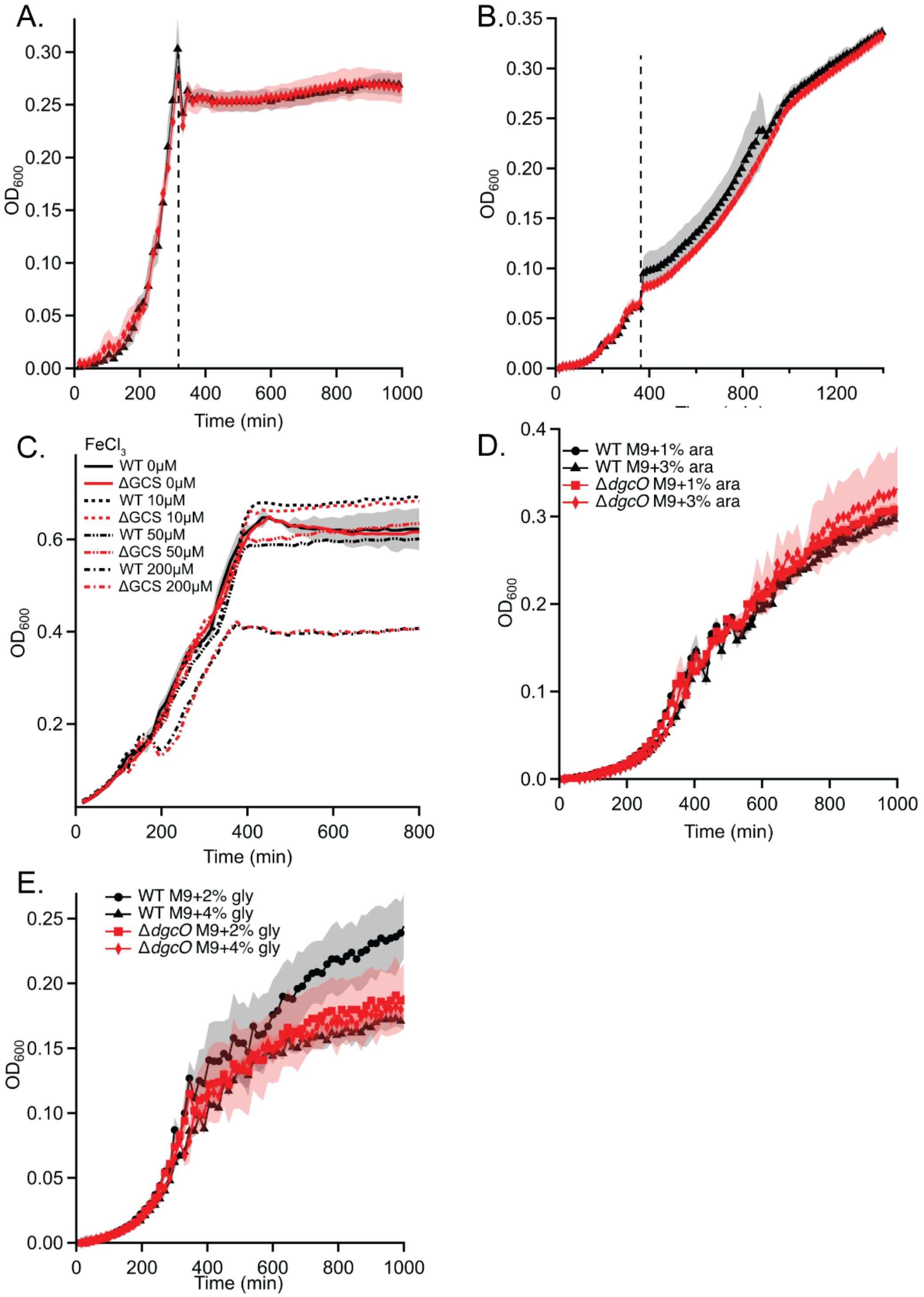

Figure S1. Growth curves of *Pcc* WPP14 WT and Δ*dgcO*. **A**) Strains grown aerobically in LB and then transitioned to anaerobic growth at the dashed line. **B**) Strains grown anaerobically in LB and then transitioned to aerobic growth at the dashed line. **C**) Strains grown in M9+glucose with various concentrations of $FeCl_3$. **D**) Strains grown in M9 with arabinose. **E**) Strains grown in M9 with arabinose.

Scar sequence from *Pcc* WPP Δ*dgcO*

Sequence between bp. 1782164 and bp. 1783158 of the published *Pcc* WPP14 genome under NCBI GenBank accession number CP051652.1 with the primary assembly GCF_013488025 (Liu et al., 2020)

CACTCGCGCGTTGCGACTTTAAGTTATCCAGCCATTTGTCAGCCCCTACCCGGCCGAACGCTC
TGGCCGCCAAGGGACGTTCATCGCTCGCCCTTCCCCGCCAGGCCAATACGGAAAACGGTAAC
TGGGAAACGTTCTAATACTGGATACCAACGTCACTGCGAGCATTGTTCACTCGGACAATGCT
CGCATATTGGCATCATCCATTTACTCTTTTTCTGGCAATATCTTTGGTTCAACAAACGTACCG
TCTTTGCTATCAACCTCATTGAAAAACCAGATGCCAGCTGGGTAATCTTTTAATGCAACTAA
ATACATTATCCCTTCCTGAAACGTATCTACAGCCAGAATCGTCCCTTCGCGGCGCGTCTTCCC
ATCGGTTTTAACCGTAACCAGATCATTAACTTTCATAAATTTATCTCAAAATAGCTTCTAACA
TCAAAAAAACAGCATTATCATTTACAACTACGGTAGGAATAGTACCTACATGAAATGATTAA
TTTTATTTTTAACAACGCCATGTTGACCCTCTCTGTCATTACATATCAATTATTCAGGCAAAA
ATTGATTGTTTCTATCCTAGTACATCCGACAT-ATTCGC-
AAAAGAGATTAAAAGTTGAACGAACTATTGAACGTTAATGAACAGAGAGATAACGGTGAGT
CAGACTACAATCAAGTCATAACATCAGAATGGATGCAATTGATAGCCACAACCTCACAAAA
ATCTTTTAATCTACTGCGAACCCTCGCCGATCAGAAAGCCAACGGCAGAAACAGGATCGAAT
ACGCGCCGGAATAACTATTCTATCTWATTGCAAACCTGCGTTTCAGTTCACATCTGAAAAGA
ATAGCGCCCTATTCATCATCGCAGGTTTACACCATATTCCTTACCAAACAGATGAATTTCCCT
CCATTGAACAAATTCACAGCATGAAAACGATAAACCAACGATTTTACTAACCAACCCGCTAG
AAATTTGATACATATTCAATAAATAAGCATGATCGTCAACAATGCGTCATGCAGTAAATCTT
TTATACTTTGATCTTATTTTATCCTGGGG-
AGGAACGAGCATTGCAGCGACAGCAGGTTTCATCGTCACGAATTGATTCAATTGGGTATGAC
CCAAAGACGCACACGCTTGAGATAGCATTTCATAACAAAGATATCTATCAGTATGTCGGTGT
GCCTGAATCTATCTATAAAAAGTTTATTTCTGACGCTGTCGTCTCTAAGGGCCGTTTCTTTGA
TGGCGTGATAAAAGACAAATTCCTATGCCGAAAAAACAAGTAGGTAAAAATCTAGCGGCAG
CCGTTTCGCTACTTTTCGCCGTCTCTTAACGGCGAGTAAAAGAGTGGCTTTACACCTCATAAT
TTGCCATTTCATCCACCAGCCCTGCCTTCCAAAATCGCAACTAATCATATGAATTAAATATAA
ATGGCGTCATGTAGCGATGTCGGTTAATAACGCGCATCGTTCGTTATACTACAAGAACACTT
GCAGGTGATGTTCCCCTTTCTCTAGCACAATTCCGACCTGTTTCTTCTCTGCATCGAATAGCA
TATACGCCATCCAGTATGC

Table S1. Transcripts found to be differentially expressed between WT and Δ*dgcO Pcc*, sorted by cellular function. Log_2FC relative to WT.

LOCUS TAG	GENE NAME	DESCRIPTION	log_2FC
Iron homeostasis			
HER17_19720	exbB	tol-pal system-associated acyl-CoA thioesterase	1.34
HER17_19715	exbD	TonB system transport protein ExbD	1.26
HER17_11795	ftnA	non-heme ferritin	-1.36
HER17_15135	fur	ferric iron uptake transcriptional regulator	-1.36
HER17_09095		heme lyase CcmF/NrfE family subunit	1.10
HER17_06780		iron ABC transporter permease	1.34
HER17_13385		iron ABC transporter permease	1.08
HER17_12160		iron chelate uptake ABC transporter family permease subunit	1.06
HER17_14235		iron transporter	1.26
HER17_02065	bfr	bacterioferritin	-1.15
HER17_09090	ccmE	cytochrome c maturation protein CcmE	1.07
HER17_13795		cyclic peptide export ABC transporter	-1.06
HER17_14240		FTR1 family iron permease	1.49
Methyl accepting chemotaxis proteins			
HER17_19330		HAMP domain-containing protein	1.20
HER17_12245		HAMP domain-containing protein	1.16
HER17_21165		HAMP domain-containing protein	1.00
HER17_19320		methyl-accepting chemotaxis protein	1.65
HER17_10660		methyl-accepting chemotaxis protein	1.21
HER17_15125		methyl-accepting chemotaxis protein	1.02
Ribosomal proteins			
HER17_02115	rpmC	50S ribosomal protein L29	-1.03
HER17_11550	rpmI	50S ribosomal protein L35	-1.41
HER17_02180	rpmJ	50S ribosomal protein L36	-1.17
HER17_02070	rpsJ	30S ribosomal protein S10	-1.09
HER17_02185	rpsM	30S ribosomal protein S13	-1.01
HER17_02120	rpsQ	30S ribosomal protein S17	-1.20
Stress response			
HER17_02560	ahpF	alkyl hydroperoxide reductase subunit F	1.17
HER17_12445	clpS	ATP-dependent Clp protease adapter ClpS	-1.19
HER17_09300	gloA	lactoylglutathione lyase	-1.26
HER17_00180	ibpA	heat shock chaperone IbpA	1.47

HER17_00185	ibpB	heat shock chaperone IbpB	1.11
HER17_07715	iraP	anti-adapter protein IraP	-1.30
HER17_11275	mgtS	protein MgtS	-1.11
HER17_02215	mscL	large-conductance mechanosensitive channel protein MscL	-1.18
HER17_02860	nhaA	Na+/H+ antiporter NhaA	1.47
HER17_09435	yciH	stress response translation initiation inhibitor YciH	1.01
HER17_15345	ypfM	protein YpfM	-1.74
HER17_03935	ytfE	iron-sulfur cluster repair protein YtfE	-1.62
HER17_11320		Slp family lipoprotein	1.01
HER17_16940		VOC family protein	-1.01
Carbohydrate metabolism			
HER17_09445	araD	L-ribulose-5-phosphate 4-epimerase	1.14
HER17_06410	galK	galactokinase	1.16
HER17_11170	gapA	glyceraldehyde-3-phosphate dehydrogenase	-1.08
HER17_04100	garL	2-dehydro-3-deoxyglucarate aldolase	1.17
HER17_04105	garR	2-hydroxy-3-oxopropionate reductase	1.20
HER17_14585	gmd	GDP-mannose 4,6-dehydratase	-1.08
HER17_04095	gudD	glucarate dehydratase	1.45
HER17_12230		2-hydroxycarboxylate transporter family protein	1.87
HER17_18000		alpha-galactosidase	1.14
HER17_15685		exo-poly-alpha-D-galacturonosidase	1.59
HER17_04090		glucarate dehydratase	1.26
HER17_00160		glycoside hydrolase family 1 protein	1.05
HER17_04110	garK	glycerate kinase	1.14
HER17_14070		helix-turn-helix transcriptional regulator	1.32
HER17_09905		SDR family oxidoreductase	-1.18
Transporters			
HER17_21760	benE	benzoate/H(+) symporter BenE family transporter	-1.37
HER17_15670	copA	copper-exporting P-type ATPase CopA	1.03
HER17_00195		anion permease	1.53
HER17_03040	thiP	thiamine/thiamine pyrophosphate ABC transporter permease ThiP	1.19
HER17_06390	ugpC	sn-glycerol-3-phosphate ABC transporter ATP-binding protein UgpC	1.18
HER17_03525	ugpC	sn-glycerol-3-phosphate ABC transporter ATP-binding protein UgpC	1.07
HER17_11845	znuA	zinc ABC transporter substrate-binding protein ZnuA	-1.05
HER17_14220	lolE	ABC transporter permease	1.15
HER17_14225	SalY	ABC transporter permease	1.57

HER17_09225		ABC transporter permease subunit	1.27
HER17_06775		ABC transporter substrate-binding protein	1.60
HER17_14135		anaerobic C4-dicarboxylate transporter	1.54
HER17_16870		capsular biosynthesis protein	-1.23
HER17_19695	LamB	carbohydrate porin	1.02
HER17_06395		maltoporin	1.26
HER17_04085		MFS transporter	1.45
HER17_21120		sugar ABC transporter permease	1.17
HER17_06380		sugar ABC transporter permease	1.03
HER17_03530		sugar ABC transporter permease	1.01
HER17_14725		sugar ABC transporter substrate-binding protein	-1.07
HER17_06820		sugar-binding protein	-1.09
Cofactor biosynthesis			
HER17_20545	coaA	type I pantothenate kinase	-1.25
HER17_13435	moaD	molybdopterin synthase sulfur carrier subunit	1.38
HER17_13430	moaE	molybdopterin synthase catalytic subunit MoaE	1.04
HER17_01995		YggS family pyridoxal phosphate-dependent enzyme	3.33
Flagella			
HER17_08120	flhC	flagellar transcriptional regulator FlhC	-1.02
HER17_08115	flhD	flagellar transcriptional regulator FlhD	-1.68
HER17_08295	fliJ	flagella biosynthesis chaperone FliJ	-1.15
HER17_08285	fliL	flagellar basal body-associated protein FliL	-1.28
HER17_08280	fliM	flagellar motor switch protein FliM	-1.05
HER17_08265	fliP	flagellar type III secretion system pore protein FliP	-1.15
HER17_08395	fliZ	flagella biosynthesis regulatory protein FliZ	-1.91
HER17_16260		flagella biosynthesis regulatory protein FliZ	-1.88
Citrate homeostasis			
HER17_12220	citC	[citrate (pro-3S)-lyase] ligase	1.88
HER17_12215	citD	citrate lyase acyl carrier protein	1.68
HER17_12210	citE	citrate (pro-3S)-lyase subunit beta	2.00
HER17_12205	citF	citrate lyase subunit alpha	1.68
HER17_12200	citX	citrate lyase holo-[acyl-carrier protein] synthase	1.79
Type VI secretion system			
HER17_04845	tssB	type VI secretion system contractile sheath small subunit	-1.30
HER17_04850	tssC	type VI secretion system contractile sheath large subunit	-1.23
HER17_04855	tssE	type VI secretion system baseplate subunit TssE	-1.04

HER17_04860	tssF	type VI secretion system baseplate subunit TssF	-1.19
HER17_04865	tssG	type VI secretion system baseplate subunit TssG	-1.28
HER17_04875	tssJ	type VI secretion system lipoprotein TssJ	-1.09
HER17_18070		Hcp family type VI secretion system effector	-2.58
HER17_16255		Hcp family type VI secretion system effector	-2.29
HER17_03625		Hcp family type VI secretion system effector	-2.18
HER17_20185		Hcp family type VI secretion system effector	-1.95
HER17_07680		Hcp family type VI secretion system effector	-1.85
HER17_20770		Hcp family type VI secretion system effector	-1.78
HER17_00070		Hcp family type VI secretion system effector	-1.66
HER17_04825		Hcp family type VI secretion system effector	-1.55
HER17_13485		Hcp family type VI secretion system effector	-1.06
Type II Toxin-Antitoxin system			
HER17_00610		type II toxin-antitoxin system RelE/ParE family toxin	-1.60
HER17_00585		type II toxin-antitoxin system RelE/ParE family toxin	-1.23
HER17_14430		type II toxin-antitoxin system RelE/ParE family toxin	-1.14
HER17_19465		type II toxin-antitoxin system RelE/ParE family toxin	-1.12
HER17_19630		type II toxin-antitoxin system RelE/ParE family toxin	-1.11
HER17_15805	hha	hemolysin expression modulator Hha	-1.36
HER17_04790		addiction module toxin RelE	-1.13
Phosphotransferase PTS system			
HER17_11220		PTS sugar transporter subunit IIB	-1.75
HER17_00150		PTS sugar transporter subunit IIB	2.08
HER17_03755		PTS sugar transporter subunit IIB	1.13
HER17_00155		PTS sugar transporter subunit IIC	1.31
HER17_11235		PTS transporter subunit EIIA	-1.11
HER17_17225	ptsH	phosphocarrier protein Hpr	-1.02
HER17_03055		glucose uptake inhibitor SgrT	1.82
Glycosyltransferases			
HER17_14615		glycosyltransferase	-2.09
HER17_14590		glycosyltransferase	-1.31
HER17_14600		glycosyltransferase family 4 protein	-1.17
HER17_16835		glycosyltransferase family 4 protein	-1.15
HER17_14595		glycosyltransferase family 4 protein	-1.05
Biofilm			
HER17_08620	bssS	biofilm formation regulator BssS	-1.21
HER17_16780	bcsQ	ParA family protein	-1.85

Anti-phage defense			
HER17_19645	cas1e	type I-E CRISPR-associated endonuclease Cas1	1.02
HER17_19615	cas2e	type I-E CRISPR-associated endoribonuclease Cas2	-1.62
HER17_19655	cas5e	type I-E CRISPR-associated protein Cas5/CasD	1.35
HER17_19650	cas6e	type I-E CRISPR-associated protein Cas6/Cse3/CasE	1.01
HER17_19660	cas7e	type I-E CRISPR-associated protein Cas7/Cse4/CasC	1.12
HER17_10475		RNA-directed DNA polymerase	-1.87
Amino acid synthesis			
HER17_21640	asnA	aspartate-ammonia ligase	1.56
HER17_15165	asnB	asparagine synthase B	1.50
HER17_19405	aspA	aspartate ammonia-lyase	1.67
Cell division			
HER17_14900	cpoB	cell division protein CpoB	-1.01
HER17_19850	parE	DNA topoisomerase IV subunit B	1.03
HER17_01600		ABC transporter ATP-binding protein	1.22
HER17_06505	ppiB	peptidylprolyl isomerase B	-1.04
Respiration			
HER17_07310	cydB	cytochrome d ubiquinol oxidase subunit II	1.28
HER17_14720	fdnI	formate dehydrogenase-N subunit gamma	-1.15
HER17_02385	frdD	fumarate reductase subunit FrdD	1.60
HER17_01445	glpB	glycerol-3-phosphate dehydrogenase subunit GlpB	2.87
HER17_01450	glpC	anaerobic glycerol-3-phosphate dehydrogenase subunit C	2.38
HER17_08615		cytochrome b	-1.35
HER17_20230		oxidoreductase	1.36
Pyrimidine metabolism/synthesis			
HER17_18180	deoA	thymidine phosphorylase	1.15
HER17_20900	pyrE	orotate phosphoribosyltransferase	1.61
HER17_20715	udp	uridine phosphorylase	1.83
Translation regulation			
HER17_20115	hpf	ribosome hibernation promoting factor	-1.12
HER17_11555	infC	translation initiation factor IF-3	-1.03
Nitrogen metabolism			
HER17_09120	napB	nitrate reductase cytochrome c-type subunit	1.01
HER17_09130	napG	ferredoxin-type protein NapG	1.09
HER17_07360	nirD	nitrite reductase small subunit NirD	1.71

Transcription			
HER17_02865	nhaR	transcriptional activator NhaR	1.09
HER17_19295	rhaS	HTH-type transcriptional activator RhaS	1.03
HER17_10865	ydgT	transcription modulator YdgT	-1.38
HER17_10060	rpoE	RNA polymerase sigma factor	-1.07
HER17_08465		TfoX/Sxy family DNA transformation protein	-1.20
HER17_08065		transcriptional regulator	-1.33
Aromatic compound metabolism			
HER17_10715	pptA	tautomerase PptA	-1.24
HER17_12225		fumarylacetoacetate hydrolase family protein	2.07
Carbohydrate biosynthesis			
HER17_14640	rfbA	glucose-1-phosphate thymidylyltransferase RfbA	-1.53
HER17_14635		WxcM-like domain-containing protein	-1.79
Type III secretion system			
HER17_10040	sctN	type III secretion system ATPase SctN	1.05
tRNA synthesis			
HER17_07015	truA	tRNA pseudouridine(38-40) synthase TruA	1.48
Antibiotic resistance			
HER17_14795		multidrug efflux pump-associated protein, AcrZ family	1.08
HER17_06630		TauD/TfdA family dioxygenase	-1.76
HER17_07460		TetR/AcrR family transcriptional regulator	-1.43
HER17_14360	acrR	TetR/AcrR family transcriptional regulator	1.09
HER17_08610		YceI family protein	-1.68
Competition, virulence			
HER17_12230		2-hydroxycarboxylate transporter family protein	1.87
HER17_10630		Expansin-YoaJ	-1.19
HER17_14280		pectate lyase	-1.33
HER17_16180		pectate lyase	-1.26
HER17_01905		polysaccharide lyase	-1.04
HER17_08360	wbqC	WbqC family protein	-2.39
c-di-GMP signaling			
HER17_13830		diguanylate cyclase	-1.47
HER17_11910	yuxH	EAL domain-containing protein	-1.62
Quorum sensing			
HER17_13960		LuxR family transcriptional regulator	-1.74

HER17_02680		LuxR family transcriptional regulator	-1.21
HER17_21095	expR	LuxR family transcriptional regulator ExpR	-1.38
Phage genes			
HER17_18670		phage repressor protein CI	-1.62
HER17_07825		phage tail protein I	1.95
HER17_04730		tyrosine-type recombinase/integrase	-1.02

Table S2. The 100 transcripts with the greatest differential expression for *Pcc* Δ*dgcO* grown under aerobic vs. anaerobic conditions. The observed changes in *Pcc* WT are included for comparison. Log_2FC relative to aerobic conditions. Log_2FC values highlighted in red exhibit the opposite regulation in *Pcc* Δ*dgcO* and WT.

LOCUS TAG	GENE NAME	DESCRIPTION	log_2FC Δ*dgcO*	log_2FC WT
HER17_02555	ahpC	alkyl hydroperoxide reductase subunit C	6.20	6.56
HER17_02560	ahpF	alkyl hydroperoxide reductase subunit F	5.94	6.58
HER17_11775		hypothetical protein	5.02	5.42
HER17_01450	glpC	anaerobic glycerol-3-phosphate dehydrogenase subunit C	4.47	5.19
HER17_04450	trxC	thioredoxin TrxC	3.91	3.75
HER17_01445	glpB	glycerol-3-phosphate dehydrogenase subunit GlpB	3.85	4.58
HER17_09860		threo-3-hydroxy-L-aspartate ammonia-lyase	3.58	3.67
HER17_09475		FdhF/YdeP family oxidoreductase	3.47	5.24
HER17_09855		ornithine cyclodeaminase family protein	3.38	3.40
HER17_11780		hypothetical protein	3.29	2.89
HER17_09850		DSD1 family PLP-dependent enzyme	3.18	3.23
HER17_01065	ilvL	ilv operon leader peptide	3.17	3.09
HER17_15855		PLP-dependent cysteine synthase family protein	3.07	3.18
HER17_12985		hypothetical protein	3.07	3.69
HER17_09790		DUF481 domain-containing protein	2.80	2.71
HER17_14865	nadA	quinolinate synthase NadA	2.68	3.31
HER17_01070		acetolactate synthase 2 catalytic subunit	2.67	2.81
HER17_12820		methyl-accepting chemotaxis protein	2.66	3.27
HER17_06555		4-deoxy-4-formamido-L-arabinose-phosphoundecaprenol deformylase	2.61	2.59
HER17_01075	ilvM	acetolactate synthase 2 small subunit	2.56	2.33
HER17_12565		GrxA family glutaredoxin	2.51	1.56
HER17_06425		organic hydroperoxide resistance protein	2.47	3.59

HER17_12825		hypothetical protein	2.41	2.69
HER17_16885		hypothetical protein	2.35	1.53
HER17_06935		spore coat protein U domain-containing protein	2.33	2.34
HER17_18305		DUF2589 domain-containing protein	2.32	3.18
HER17_06560	arnT	lipid IV(A) 4-amino-4-deoxy-L-arabinosyltransferase	2.30	2.59
HER17_06420		MarR family transcriptional regulator	2.28	2.05
HER17_15070	kdpF	K(+)-transporting ATPase subunit F	2.28	2.32
HER17_06565		EamA family transporter	2.25	1.97
HER17_06550	arnA	bifunctional UDP-4-amino-4-deoxy-L-arabinose formyltransferase/UDP-glucuronic acid oxidase ArnA	2.25	2.22
HER17_15575		catalase	2.21	1.20
HER17_08575		PepSY domain-containing protein	2.20	2.60
HER17_06190		hypothetical protein	2.18	1.19
HER17_18230	yrbN	protein YrbN	2.18	2.40
HER17_03300	aceE	pyruvate dehydrogenase (acetyl-transferring), homodimeric type	2.17	2.97
HER17_18320		hypothetical protein	2.15	1.14
HER17_17590		hypothetical protein	2.14	2.37
HER17_14080		HAMP domain-containing protein	2.12	2.30
HER17_01520	glpD	glycerol-3-phosphate dehydrogenase	2.10	2.36
HER17_15075	kdpA	potassium-transporting ATPase subunit KdpA	2.10	-1.40
HER17_13520		CidA/LrgA family protein	2.07	-2.28
HER17_00765		energy transducer TonB	2.06	-1.85
HER17_06940		spore coat protein U domain-containing protein	2.06	-1.54
HER17_03295	pdhR	pyruvate dehydrogenase complex transcriptional repressor PdhR	2.05	-2.95
HER17_08060		restriction endonuclease	2.04	-3.18
HER17_03305	aceF	pyruvate dehydrogenase complex dihydrolipoyllysine-residue acetyltransferase	2.02	-2.53
HER17_06185		hypothetical protein	2.01	-1.88
HER17_00835	glpK	glycerol kinase GlpK	2.00	-2.45
HER17_20230		oxidoreductase	-2.31	-2.34
HER17_14445	iolC	5-dehydro-2-deoxygluconokinase	-2.32	-2.38
HER17_01995		YggS family pyridoxal phosphate-dependent enzyme	-2.34	-1.87
HER17_11865		PTS cellobiose transporter subunit IIC	-2.35	-1.71
HER17_19645	cas1e	type I-E CRISPR-associated endonuclease Cas1	-2.35	-2.88

HER17_09895		sulfotransferase family protein	-2.36	-2.37
HER17_09890		aspartate aminotransferase family protein	-2.36	-2.67
HER17_17340		oxidoreductase	-2.37	-1.57
HER17_00195		anion permease	-2.37	-3.05
HER17_10775		ROK family transcriptional regulator	-2.38	-3.45
HER17_04880	tssK	type VI secretion system baseplate subunit TssK	-2.39	-4.05
HER17_03545		GNAT family N-acetyltransferase	-2.40	-2.85
HER17_04870	tagH	type VI secretion system-associated FHA domain protein TagH	-2.43	-2.69
HER17_20735	metE	5-methyltetrahydropteroyltriglutamate--homocysteine S-methyltransferase	-2.44	-2.17
HER17_20900	pyrE	orotate phosphoribosyltransferase	-2.50	-2.47
HER17_03740		PTS lactose/cellobiose transporter subunit IIA	-2.51	-2.65
HER17_09005	bglG	transcriptional antiterminator BglG	-2.51	-2.27
HER17_01635		methyl-accepting chemotaxis protein	-2.52	-1.74
HER17_07125		YfbU family protein	-2.53	-1.99
HER17_03375		YacL family protein	-2.53	-2.26
HER17_01720	pckA	phosphoenolpyruvate carboxykinase (ATP)	-2.54	-2.20
HER17_00150		PTS sugar transporter subunit IIB	-2.56	-2.70
HER17_09885		2OG-Fe(II) oxygenase	-2.58	-2.44
HER17_10465		TIGR02646 family protein	-2.61	-3.62
HER17_15870		competence protein ComEA	-2.62	-1.99
HER17_18885	aspA	aspartate ammonia-lyase	-2.65	-2.61
HER17_01620		IclR family transcriptional regulator	-2.66	-2.80
HER17_04915		hypothetical protein	-2.67	-3.62
HER17_10375		ClbS/DfsB family four-helix bundle protein	-2.67	-2.40
HER17_04230		hypothetical protein	-2.67	-2.59
HER17_02905	carA	glutamine-hydrolyzing carbamoyl-phosphate synthase small subunit	-2.68	-2.96
HER17_18705		DUF305 domain-containing protein	-2.69	-3.91
HER17_19670	casA	type I-E CRISPR-associated protein Cse1/CasA	-2.69	-3.57
HER17_08845		cupin domain-containing protein	-2.76	-2.78
HER17_04900	tagO	type VI secretion system-associated protein TagO	-2.78	-2.71
HER17_04920		sel1 repeat family protein	-2.81	3.18
HER17_03975		DUF1107 domain-containing protein	-2.81	3.69
HER17_19560	pyrB	aspartate carbamoyltransferase	-2.83	2.71
HER17_00045		PTS sugar transporter subunit IIB	-2.89	3.31
HER17_16945		hypothetical protein	-2.93	2.81

HER17_05175		phage tail sheath protein	-2.94	3.27
HER17_04895		sigma-54-dependent Fis family transcriptional regulator	-2.95	2.59
HER17_04735		hypothetical protein	-2.96	2.33
HER17_04885		DotU family type IV/VI secretion system protein	-2.96	1.56
HER17_04910	tssM	type VI secretion system membrane subunit TssM	-3.02	3.59
HER17_04890	tssH	type VI secretion system ATPase TssH	-3.07	2.69
HER17_21870		sce7725 family protein	-3.07	1.53
HER17_21865		RES family NAD+ phosphorylase	-3.12	2.34
HER17_08850		class I SAM-dependent methyltransferase	-3.26	3.18
HER17_00190		putative transporter	-3.37	2.59
HER17_04905	tssA	type VI secretion system protein TssA	-3.76	2.05

Reference

LIU, Y., HELMANN, T. C., STODGHILL, P. & FILIATRAULT, M. J. 2020. Complete Genome Sequence of the Necrotrophic Plant-Pathogenic Bacterium Pectobacterium carotovorum WPP14. Plant Pathology, Cornell University.
